# A wind power plant site selection algorithm based on *q*-rung orthopair hesitant fuzzy rough Einstein aggregation information

**DOI:** 10.1038/s41598-022-09323-5

**Published:** 2022-03-31

**Authors:** Attaullah, Shahzaib Ashraf, Noor Rehman, Asghar Khan, Muhammad Naeem, Choonkil Park

**Affiliations:** 1 Department of Mathematics, Abdul Wali Khan University, Mardan, KPK 23200 Pakistan; 2 Department of Mathematics, Khawaja Farid University of Engineering and Information Technology, Rahim Yar Khan, Pakistan; 3 Department of Mathematics and Statistics, Bacha Khan University, Charsadda, KPK Pakistan; 4 Deanship of Combined First Year, Umm Al-Qura University, Makkah, Saudi Arabia; 5 Research Institute for Natural Sciences, Hanyang University, Seoul, Korea

**Keywords:** Environmental social sciences, Energy science and technology, Mathematics and computing

## Abstract

Wind power is often recognized as one of the best clean energy solutions due to its widespread availability, low environmental impact, and great cost-effectiveness. The successful design of optimal wind power sites to create power is one of the most vital concerns in the exploitation of wind farms. Wind energy site selection is determined by the rules and standards of environmentally sustainable development, leading to a low, renewable energy source that is cost effective and contributes to global advancement. The major contribution of this research is a comprehensive analysis of information for the multi-attribute decision-making (MADM) approach and evaluation of ideal site selection for wind power plants employing *q*-rung orthopair hesitant fuzzy rough Einstein aggregation operators. A MADM technique is then developed using *q*-rung orthopair hesitant fuzzy rough aggregation operators. For further validation of the potential of the suggested method, a real case study on wind power plant site has been given. A comparison analysis based on the unique extended TOPSIS approach is presented to illustrate the offered method’s capability. The results show that this method has a larger space for presenting information, is more flexible in its use, and produces more consistent evaluation results. This research is a comprehensive collection of information that should be considered when choosing the optimum site for wind projects.

## Introduction

Providing sustainable and widely accessible energy to human populations became one of the most challenging problems over the last several decades. Between 2000 and 2030 AD, global energy consumption is expected to expand by an average of 8% each year^1^. Fossil fuels supply the majority of the energy needed and have the largest effect. To reduce their reliance on fossil fuels, several developed nations have enacted laws promoting the use of renewable energy sources such as wind and solar power. Wind power is one of the most reliable and long-term renewable energy sources accessible. Wind energy has developed into a large, environmentally beneficial, and financially feasible resource. It has become more desirable as a renewable resource attributable to technological improvements and productivity improvements. Wind energy has grown in popularity, and governments have implemented several successful policies that encourage its installation. The expense of wind energy generation has become comparable with the cost of fossil fuel generation. As a result, wind energy is a surprisingly safe and risk-free source of renewable energy that is economically feasible, ecologically safe, and contributed substantially to the reduction of hazardous substances. The main goal of the study is to assess the requirement for excess power resources as a consequence of growing populations. Prior to initiating the technical project, it is essential to choose the suitable location for the wind power plant. The enhancement of facility location provides relevant information of analyzing challenges associated with installations in specific locations based on specified criteria^2,3^. Wind farm site selection is complicated, with many factors to consider, including the finance, environment, infrastructure, ecological, geographical features, hydrogeological engineering conditions, ground hydrological conditions, industry, and practicality of wind power^4,5^.

Owing to the overwhelming ambiguity and complexity of local and global surroundings, as well as the capacity of human intelligence to apprehend reality, it is not always possible for decision makers to express assessment values or ideas in simple quantitative measures. To alleviate this challenge, Zadeh^83^ established the fuzzy set theory, which is characterized by a membership function with a degree of membership of [0,1]. In recent decades, fuzzy set theories have significantly been incorporated with MCDM as a beneficial tool for resolving imprecision and ambiguity, resulting in a profusion of fuzzy MCDM techniques. However, in dealing with erroneous information caused by several sources of uncertainty in reality, the traditional fuzzy set has certain limitations. Therefore, various expansions of fuzzy sets have already been suggested over the last few decades. Torra^6,7^ introduced hesitant fuzzy sets (HFS) in 2009, which enables the membership degree to have more than one value, known as the hesitant fuzzy element (HFE), to indicate epistemic uncertainty. The HFS better captures various ambiguity in decision processes, and it may effectively express decision makers’ reticence in achieving the final consensus for one thing. Therefore, HFS is a more useful tool for representing unpredictability in MCDM. Many researchers contribute to the advancement of HFS theory and applications. For example, Xu et al.^48^ established a TOPSIS approach for MCDM problems using hesitant fuzzy information and the maximising deviation method. Liao et al.^49^ suggested various weight-determination strategies in MCDM based on HF preference information. Mahmoudi et al.^50^ expanded the PROMETHEE to an HF environment that was irrelevant to aggregation and distance operators. Alcantud et al.^51^ established HFS decomposition theorems employing newly specified families of cuts and also presented two HFS extension principles that broadened crisp maps. Following that, several innovative generalised versions of HFS are presented to effectively address ambiguity in real problems. Qian et al.^52^ developed the generalise HFS, explored its arithmetic operations and relationships with the HFS, and eventually implemented it to practical MCDM. Zhu et al.^53^ introduced dual HFS (DHFS) and analysed the basic operations and features while proposing a DHFS expansion concept. Rodriguez et al.^54,55^ addressed the HF linguistic term set (HFLT) and its applicability in group decision challenges. Chen et al.^56^ initiated internal-valued HFS (IVHFS) and provided the composite operators the IVHFS information and established a group decision framework based on the IVHF preference relations. The strategies outlined above may solve the uncertainty and vagueness in MCDM challenges. These approaches are appropriate in cases wherein decision makers may express their preferences on an alternative or a candidate using just a few possible values. However, the assessments that might be provided have equal weights or significance. Obviously, in real-world MCDM challenges, it may not be appropriate for decision makers’ hesitant judgments and assessments. In the context of the foregoing, hesitant fuzzy sets and their expansions may be able to more effectively handle unpredictable circumstances and portray expert thoughts more exhaustively and dynamically. As a consequence, a range of HFSs have been effectively used in the execution of a number of MCDM problems^57–61^. Rough set theory, introduced by Pawlak^47^, is a key mathematical tool for dealing with ambiguous, inconsistent, and incomplete data and information by recurring to the lower (higher) approximations. In MCDM, RS can better describe hidden information, which has several benefits in attribute selection, rule learning, and information interpretation. In recent years, rough set has garnered a lot of attention in both theories and applications for MCDM. Presently, RS has been broadened in a variety of different ways. A significant generalisation is to include various uncertain information processing frameworks into RS, such as fuzzy set, intuitionistic fuzzy set, and interval-valued fuzzy set, allowing for the performance of several extended models in intricate MCDM. Yang et al.^63^ made a crucial contribution to the fusion of HFSs and RSs. They established the notion of HFRSs by using an HFR. However, in Yang’s model, the ordering relationship of the HF subsets is not always antisymmetric. Zhang et al.^64^ demonstrated another HFRS approach that used a specified HF subset to address this problem. Nonetheless, many researchers developed a variety of beneficial generalized hesitant fuzzy rough set models from various perspectives, such as the HFL rough set method^65^, the IVHF RS method^66^, the dual hesitant fuzzy rough model^62^ the HF rough set model on two domains^67^, the multi granulation HF rough set model^68,69^, and so on. These approaches provide their own characteristics in many ways, such as converting one domain to two domains and relation from single to multiple^70,71^. Hussain et al.^30^ introduced a covering-based q-ROF rough set model hybrid with TOPSIS technique for MADM. In reality, these q-ROF rough set extensions have been proved to be effective in managing DM’s evaluation values in MAGDM difficulties.

Considering the limited resources accessible on planet earth, particularly in light of its numerous uses, it becomes crucial to find the most optimal location for wind power plant installation. In terms of wind energy utilisation, Bozorg et al.^88^ assessed the application of Geographical Information System (GIS) in site selection for the establishment of wind power plants in Khuzestan province, Iran. Kamau et al.^89^ estimated the Weibull parameter values using wind data from the Kenya Meteorological Department from 2001 to 2006. The findings suggested that the location was appropriate for grid-connected electricity production as well as other applications like as water pumping and battery charging. In Poland, Szkliniarz and Vogt^90^ assessed wind energy potential using a GIS-based technique. They developed a technique for employing GIS to assist in the decision-making process for wind energy project site selection. Yahyai et al.^91^ developed a wind farm site suitability index and classification in an Oman GIS environment by combining MCDM with an analytical hierarchy process (AHP) aggregation function and an Ordered Weigh Averaging (OWA) aggregation function. Rediske et al.^92^ evaluated the suitable location for wind farms and addressed the factors that restrict the choice of location. Azizi et al.^93^ explored land suitability evaluation for wind power plant site selection in the Ardabil province of Iran using analytic network process DEMATEL in a GIS environment.

Like mentioned previously, energy supply is regarded as the primary cause of discomfort in modern societies owing to the scarcity of fossil energy supplies. Thus, the deployment of renewable energy sources, particularly wind energy, is considered one of the greatest most effective administrative approaches for reducing this challenge. In recent decades, various types of approaches for the fuzzy generalizations of RS theory have been suggested and developed. Despite an abundance of achievement, the extension of FRS theory to suggest a novel information representation, which is the inspiration for this study. *q*-ROHFRS is a hybrid intelligent structure for responding with ambiguous and unclear data. Aggregation operators are critical in DM since they take data from several sources and combine it into a single value. *q*-ROHFS and rough set hybridization is not discovered in the *q*-ROF information, according to current understanding. As a consequence, we specify a collection of operators based on rough data, such as *q*-rung orthopair hesitant fuzzy rough Einstein weighted geometric, ordered weighted geometric, and hybrid weighted geometric aggregation operators.

This manuscript contributes to the literature of FRSs theory by introducing some innovative ideas, which are as follows:

(1): To compile a list of Aops based on Einstein’s t-norm and t-conorm, namely *q*-ROHFR weighted geometric, *q*-ROHFR ordered weighted geometric, and *q*-ROHFR hybrid weighted geometric operators, and explore their essential operational laws. Also, discuss the related properties thoroughly.
(2): To develop a DM approach for synthesising uncertain information utilising suggested aggregation operators.
(3): A numerical case study of a real-world problem concerning wind power project site selection evaluation is developed using the established operators.
(4): Furthermore, the findings are interpreted through comparisons to the *q*-ROHFR-TOPSIS technique. The acquired outcomes are displayed graphically.

The outliving section of the article is described as follows: “Fundamental concepts” summarises key concepts in *q*-ROFSs, HFSs, rough set theory and *q*-rung orthopair hesitant fuzzy sets. “The q-rung orthopair hesitant fuzzy rough aggregation operators” summarises a list of Einstein aggregation operators that are used to aggregate uncertain data based on Einstein operational laws. “The multi-attribute decision making methodology” explains a decision-making approach based on developed AOps. “The application of proposed decision-making approach” illustrates how to formulate a strategy for wind farm site selection numerically. Additionally, this section explores the application of the technique. “Comparison analysis” details the proposed *q*-ROHFR-TOPSIS technique for analysing the MADM method based on AOps. “Conclusion” concludes the manuscript.

## Fundamental concepts

This section introduces some fundamental concepts, particularly *q*-ROFS, RS, and *q*-ROFRS.

### Definition 2.1

(*Ref.*^15^) Let $\Im$ be a universal. A *q*-ROFS *F* over $\Im$ is define as follows:

$$\begin{aligned} F=\left\{ \langle \nu ,\eth _{F}(\nu ),\psi _{F}(\nu )\rangle |\nu \in \Im \right\} \end{aligned}$$

for each $\nu \in \Im$ the functions $\eth _{F}:\Im \rightarrow [0,~1]$ and $\psi _{F}:\Im \rightarrow [0, 1]$ denotes the positive and negative membership functions respectively with constrain that $(\psi _{F}(\nu ))^{q}+(\eth _{F}(\nu ))^{q}\le 1,(q>2\in \mathbb {Z}).$

### Definition 2.2

(*Ref.*^9^) Let $\Im$ be the universal set and $\mathcal {Y} \in q-ROFS(\Im \times \Im )$ be an IF relation. Then

(1): $\mathcal {Y}$ is reflexive if $\vartheta _{\mathcal {Y} }(\mu ,\mu )=1$ and $\delta _{\mathcal {Y} }(\mu ,\mu )=0,\forall \mu \in \Im ;$
(2): $\mathcal {Y}$ is symmetric if $\forall (\mu ,a)\in \Im \times \Im ,$ $\vartheta _{\mathcal {Y} }(\mu ,a)=\vartheta _{\mathcal {Y} }(a,\mu )$ and $\delta _{\mathcal {Y} }(\mu ,a)=\delta _{\mathcal {Y} }(a,\mu );$
(3): $\mathcal {Y}$ is transitive if $\forall (\mu ,b)\in \Im \times \Im ,$

$$\begin{aligned} \vartheta _{\mathcal {Y} }(\mu ,b)\ge \bigvee \nolimits _{a\in \Im }\left[ \vartheta _{\mathcal {Y} }(\mu ,a)\wedge \vartheta _{\mathcal {Y} }(a,b)\right] ; \end{aligned}$$

and

$$\begin{aligned} \delta _{\mathcal {Y} }(\mu ,b)=\bigwedge \nolimits _{a\in \Im }\left[ \delta _{\mathcal {Y} }(\mu ,a)\wedge \delta _{\mathcal {Y} }(a,b)\right] . \end{aligned}$$

### Definition 2.3

Let $\Im$ be the universal set. Then any $\mathcal {Y} \in q-RFS(\Im \times \Im )$ is called *q*-rung relation. The pair $\left( \Im , \mathcal {Y} \right)$ is said to be a *q*-rung approximation space. Now for any $\mathcal {B} \subseteq q-RFS(\Im )$, the upper and lower approximations of $\mathcal {B}$ with respect to *q*-rung fuzzy approximation space $\left( \Im ,\mathcal {Y} \right)$ are two *q*-RFSs, which are denoted by $\overline{\mathcal {Y} }(\mathcal {B} )$ and $\underline{\mathcal {Y} }(\mathcal {B} )$ and is defined as:

$$\begin{aligned} \overline{\mathcal {Y} }(\mathcal {B} )= & {} \left\{ \left\langle \mu ,\vartheta _{\overline{\mathcal {Y} }(\mathcal {B} )}(\mu ),\delta _{\overline{\mathcal {Y} }(\mathcal {B} )}(\mu )\right\rangle |\mu \in \Im \right\} ; \\ \underline{\mathcal {Y} }(\mathcal {B} )= & {} \left\{ \left\langle \mu ,\vartheta _{\underline{ \mathcal {Y} }(\mathcal {B} )}(\mu ),\delta _{\underline{\mathcal {Y} }(\mathcal {B} )}(\mu )\right\rangle |\mu \in \Im \right\} ; \end{aligned}$$

where

$$\begin{aligned} \vartheta _{\overline{\mathcal {Y} }(\mathcal {B} )}(\mu )= & {} \underset{g\in \Im }{ \bigvee }[\vartheta _{\mathcal {Y} }(\mu ,g)\bigvee \vartheta _{\mathcal {B} }(g)]; \\ \delta _{\overline{\mathcal {Y} }(\mathcal {B} )}(\mu )= & {} \underset{g\in \Im }{ \bigwedge }[\delta _{\mathcal {Y} }(\mu ,c)\bigwedge \delta _{\mathcal {B} }(g)]; \\ \vartheta _{\underline{\mathcal {Y} }(\mathcal {B} )}(\mu )= & {} \underset{g\in \Im }{ \bigwedge }[\vartheta _{\mathcal {Y} }(\mu ,c)\bigwedge \vartheta _{\mathcal {B} }(g)]; \\ \delta _{\underline{\mathcal {Y} }(\mathcal {B} )}(\mu )= & {} \underset{g\in \Im }{ \bigvee }[\delta _{\mathcal {Y} }(\mu ,c)\bigvee \delta _{\mathcal {B} }(g)]; \end{aligned}$$

such that $0\le ((\vartheta _{\overline{\mathcal {Y} }(\mathcal {B} )}(\mu ))^{q}+(\delta _{\overline{\mathcal {Y} }(\mathcal {B} )}(\mu ))^{q})\le 1,$ and $0\le \left( \left( \vartheta _{\underline{\mathcal {Y} }(\mathcal {B} )}(\mu )\right) ^{q}+\left( \delta _{\underline{\mathcal {Y} }(\mathcal {B} )}(\mu )\right) ^{q}\right) \le 1.$ As $\left( \underline{\mathcal {Y} }(\mathcal {B} ),\overline{ \mathcal {Y} }(\mathcal {B} )\right)$ are $q-RFSs,$ so $\underline{\mathcal {Y} }(\mathcal {B} ),$ $\overline{\mathcal {Y} }(\mathcal {B} ):q-RFS(\Im )\rightarrow q-RFS(\Im )$ are upper and lower approximation operators. The pair $\mathcal {Y} (\mathcal {B} )=( \underline{\mathcal {Y} }(\mathcal {B} ),\overline{\mathcal {Y} }(\mathcal {B} ))=\{\left\langle \mu ,(\vartheta _{\underline{\mathcal {Y} }(\mathcal {B} )}(\mu ),\delta _{\underline{\mathcal {Y} } (\mathcal {B} )}(\mu ),(\vartheta _{\overline{\mathcal {Y} }(\mathcal {B} )}(\mu ),\delta _{ \overline{\mathcal {Y} }(\mathcal {B} )}(\mu ))\right\rangle |\mu \in \mathcal {B} \}$ is known as q-rung rough set. For simplicity $\mathcal {Y} (\mathcal {B} )=\{\left\langle \mu ,\vartheta _{\underline{\mathcal {Y} }(\mathcal {B} )}(\mu ),\delta _{ \underline{\mathcal {Y} }(\mathcal {B} )}(\mu ),(\vartheta _{\overline{\mathcal {Y} }(\mathcal {B} )}(\mu ),\delta _{\overline{\mathcal {Y} }(\mathcal {B} )}(\mu ))\right\rangle |\mu \in \Im \}$ is represented as $\mathcal {Y} (\mathcal {B} )=((\underline{\vartheta }, \underline{\delta }),(\overline{\vartheta },\overline{\delta }))$ and is known as *q*-RFRV.

### Definition 2.4

^34^ Let $\Im$ be a nonempty finite set and for any subset $\mathcal {Y} \in q-ROHFS(\Im \times \Im )$ is said to be a *q*-rung hesitant fuzzy relation. The pair $\left( \Im ,\mathcal {Y} \right)$ is said to be *q*-ROHF approximation space. If for any $\mathcal {B} \subseteq q-ROHFS(\Im )$, then the upper and lower approximations of $\mathcal {B}$ with respect to *q*-ROHF approximation space $\left( \Im ,\mathcal {Y} \right)$ are two *q*-ROHFSs, which are denoted by $\overline{\mathcal {Y} }(\mathcal {B} )$ and $\underline{\mathcal {Y} } (\mathcal {B} )$ and defined as:

$$\begin{aligned} \overline{\mathcal {Y} }(\mathcal {B} )= & {} \left\{ \left\langle \mu ,\eth _{h_{ \overline{\mathcal {Y} }(\mathcal {B} )}}(\mu ),\psi _{h_{\overline{\mathcal {Y} }(\mathcal {B} )}}(\mu )\right\rangle |\mu \in \Im \right\} ; \\ \underline{\mathcal {Y} }(\mathcal {B} )= & {} \left\{ \left\langle \mu ,\eth _{h_{ \underline{\mathcal {Y} }(\mathcal {B} )}}(\mu ),\psi _{h_{\underline{\mathcal {Y} } (\mathcal {B} )}}(\mu )\right\rangle |\mu \in \Im \right\} ; \end{aligned}$$

where

$$\begin{aligned} \eth _{h_{\overline{\mathcal {Y} }(\mathcal {B} )}}(\mu )= & {} \underset{k\in \Im }{ \bigvee }\left[ \eth _{h_{\mathcal {Y} }}(\mu ,k)\bigvee \eth _{h_{\mathcal {B} }}(k)\right] ; \\ \psi _{h_{\overline{\mathcal {Y} }(\mathcal {B} )}}(\mu )= & {} \underset{k\in \Im }{ \bigwedge }\left[ \psi _{h_{\mathcal {Y} }}(\mu ,k)\bigwedge \psi _{h_{\mathcal {B} }}(k)\right] ; \\ \eth _{h_{\underline{\mathcal {Y} }(\mathcal {B} )}}(\mu )= & {} \underset{k\in \Im }{\bigwedge }\left[ \eth _{h_{\mathcal {Y} }}(\mu ,k)\bigwedge \eth _{h_{\mathcal {B} }}(k)\right] ; \\ \psi _{h_{\underline{\mathcal {Y} }(\mathcal {B} )}}(\mu )= & {} \underset{k\in \Im }{ \bigvee }\left[ \psi _{h_{\mathcal {Y} }}(\mu ,k)\bigvee \psi _{h_{\mathcal {B} }}(k)\right] ; \end{aligned}$$

such that $0\le \left( \max (\eth _{h_{\overline{\mathcal {Y} }(\mathcal {B} )}}(\mu ))\right) ^{q}+\left( \min (\psi _{h_{\overline{\mathcal {Y} }(\mathcal {B} )}}(\mu ))\right) ^{q}\le 1$ and $0\le \left( \min (\eth _{h_{\underline{ \mathcal {Y} }(\mathcal {B} )}}(\mu )\right) ^{q}+\left( \max (\psi _{h_{\underline{ \mathcal {Y} }(\mathcal {B} )}}(\mu ))\right) ^{q}\le 1.$ As $\left( \overline{\mathcal {Y} } (\mathcal {B} ),\underline{\mathcal {Y} }(\mathcal {B} )\right)$ are *q*-ROHFSs, so $\overline{\mathcal {Y} }(\mathcal {B} ),\underline{\mathcal {Y} }(\mathcal {B} ):q-ROHFS(\Im )\rightarrow q-RFS(\Im )$ are upper and lower approximation operators. The pair

$$\begin{aligned} \mathcal {Y} (\mathcal {B} )=\left( \underline{\mathcal {Y} }(\mathcal {B} ),\overline{\mathcal {Y} } (\mathcal {B} )\right) =\left\{ \left\langle \mu ,\left( \eth _{h_{ \underline{\mathcal {Y} }(\mathcal {B} )}}(\mu ),\psi _{h_{\underline{\mathcal {Y} } (\mathcal {B} )}}(\mu )\right) ,\left( \eth _{h_{\overline{\mathcal {Y} } (\mathcal {B} )}}(\mu ),\psi _{h_{\overline{\mathcal {Y} }(\mathcal {B} )}}(\mu )\right) \right\rangle |\mu \in \mathcal {B} \right\} \end{aligned}$$

will be called *q*-ROHFRSs. For simplicity

$$\begin{aligned} \mathcal {Y} (\mathcal {B} )=\left\{ \left\langle \mu ,\left( \eth _{h_{\underline{ \mathcal {Y} }(\mathcal {B} )}}(\mu ),\psi _{h_{\underline{\mathcal {Y} }(\mathcal {B} )}}(\mu )\right) ,\left( \eth _{h_{\overline{\mathcal {Y} }(\mathcal {B} )}}(\mu ),\psi _{h_{ \overline{\mathcal {Y} }(\mathcal {B} )}}(\mu )\right) \right\rangle |\mu \in \mathcal {B} \right\} \end{aligned}$$

is represented as $\mathcal {Y} (\mathcal {B} )=\left( (\underline{\eth },\underline{ \psi }),(\overline{\eth },\overline{\psi })\right)$ and is known as *q*-ROHFR value. We provide the following example to demonstrate the above notion of *q*-ROHFRS.

### Example 2.5

(Ref.^35^) Suppose $\Im =\left\{ \mu _{1},\mu _{2},\mu _{3},\mu _{4}\right\}$ be any arbitrary set and $\left( \Im ,\mathcal {Y} \right)$ is *q*-ROHF approximation space with $\mathcal {Y} \in q-ROHFRS(\Im \times \Im )$ be the *q* -ROHFR relation as given in Table 1. Now an expert in decision-making presents the ideal normal decision object *mathcalR*, which is a *q*-ROHFS.

**Table 1:** The *q*-ROHF relation in $\Im$.

$\Im$	$c_{1}$	$c_{2}$	$c_{3}$	$c_{4}$
$\mu _{1}$	$$\left( \begin{array}{c} \left\{ 0.1,0.3,0.4\right\} , \\ \left\{ 0.2,0.5,0.7\right\} \end{array} \right)$$	$$\left( \begin{array}{c} \left\{ 0.2,0.3\right\} , \\ \left\{ 0.7,0.9\right\} \end{array} \right)$$	$$\left( \begin{array}{c} \left\{ 0.2,0.5,0.7\right\} , \\ \left\{ 0.2,0.3\right\} \end{array} \right)$$	$$\left( \begin{array}{c} \left\{ 0.3,0.5\right\} , \\ \left\{ 0.8\right\} \end{array} \right)$$
$\mu _{2}$	$$\left( \begin{array}{c} \left\{ 0.2,0.3,0.5\right\} , \\ \left\{ 0.2,0.7\right\} \end{array} \right)$$	$$\left( \begin{array}{c} \left\{ 0.2,0.3,,0.5\right\} , \\ \left\{ 0.3,0.4\right\} \end{array} \right)$$	$$\left( \begin{array}{c} \left\{ 0.1,0.4,0.6\right\} , \\ \left\{ 0.7,0.9\right\} \end{array} \right)$$	$$\left( \begin{array}{c} \left\{ 0.2,0.4\right\} , \\ \left\{ 0.7\right\} \end{array} \right)$$
$\mu _{3}$	$$\left( \begin{array}{c} \left\{ 0.5,0.6\right\} , \\ \left\{ 0.7,0.9\right\} \end{array} \right)$$	$$\left( \begin{array}{c} \left\{ 0.5,0.8,0.9\right\} , \\ \left\{ 0.1,0.9\right\} \end{array} \right)$$	$$\left( \begin{array}{c} \left\{ 0.2,0.3\right\} , \\ \left\{ 0.5,0.9\right\} \end{array} \right)$$	$$\left( \begin{array}{c} \left\{ 0.7,0.9\right\} , \\ \left\{ 0.1,0.2,0.3\right\} \end{array} \right)$$
$\mu _{4}$	$$\left( \begin{array}{c} \left\{ 0.2,0.5,0.9\right\} , \\ \left\{ 0.6,0.7,0.9\right\} \end{array} \right)$$	$$\left( \begin{array}{c} \left\{ 0.3,0.8,0.9\right\} , \\ \left\{ 0.4,0.8\right\} \end{array} \right)$$	$$\left( \begin{array}{c} \left\{ 0.2,0.5\right\} , \\ \left\{ 0.6,0.9\right\} \end{array} \right)$$	$$\left( \begin{array}{c} \left\{ 0.5,0.7\right\} , \\ \left\{ 0.1,0.8\right\} \end{array} \right)$$

and

$$\begin{aligned} \mathcal {B} =\left\{ \begin{array}{c} \left\langle \mu _{1},\left\{ 0.2,0.3,0.4\right\} ,\left\{ 0.5,0.7\right\} \right\rangle ,\left\langle \mu _{2},\left\{ 0.2,0.3,0.7\right\} ,\left\{ 0.1,0.7,0.8\right\} \right\rangle , \\ \left\langle \mu _{3},\left\{ 0.5,0.7,0.8\right\} ,\left\{ 0.1,0.5,0.7\right\} \right\rangle ,\left\langle \mu _{4},\left\{ 0.6,0.8,0.9\right\} ,\left\{ 0.2,0.6,0.7\right\} \right\rangle \end{array} \right\} . \end{aligned}$$

Afterwards, it follows that

$$\begin{aligned} \eth _{h_{\overline{\mathcal {Y} }(\mathcal {B} )}}(\mu _{1})= & {} \underset{k\in \Im }{\bigvee }\left[ \eth _{h_{\mathcal {Y} }}(\mu ,c)\bigvee \eth _{h_{\mathcal {B} }}(k)\right] \\= & {} \left\{ \begin{array}{c} \left\{ 0.1\vee 0.2,0.3\vee 0.3,0.4\vee 0.4\right\} \vee \\ \left\{ 0.2\vee 0.2,0.3\vee 0.3,0\vee 0.7\right\} \vee \\ \left\{ 0.2\vee 0.5,0.5\vee 0.7,0.7\vee 0.8\right\} \vee \\ \left\{ 0.3\vee 0.6,0.5\vee 0.8,0\vee 0.9\right\} \end{array} \right\} \\= & {} \left\{ \begin{array}{c} \left\{ 0.2,0.3,0.4\right\} \vee \left\{ 0.2,0.3,0.7\right\} \vee \\ \left\{ 0.5,0.7,0.8\right\} \vee \left\{ 0.6,0.8,0.9\right\} \end{array} \right\} \\= & {} \left\{ 0.6,0.8,0.9\right\} . \end{aligned}$$

In a similar way, we obtain the other values:

$$\begin{aligned} \begin{array}{cc} \eth _{h_{\overline{\mathcal {Y} }(\mathcal {B} )}}(\mu _{2})=\left\{ 0.6,0.8,0.9\right\} , &{} \eth _{h_{\overline{\mathcal {Y} }(\mathcal {B} )}}(\mu _{3})=\left\{ 0.7,0.9\right\} , \\ \eth _{h_{\overline{\mathcal {Y} }(\mathcal {B} )}}(\mu _{4})=\left\{ 0.6,0.8,0.9\right\} . &{} \end{array} \end{aligned}$$

Similarly,

$$\begin{aligned} \psi _{h_{\overline{\mathcal {Y} }(\mathcal {B} )}}(\mu _{1})= & {} \underset{k\in \Im }{\bigwedge }\left[ \psi _{h_{\mathcal {Y} }}(\mu ,c)\bigwedge \psi _{h_{\mathcal {B} }}(k)\right] \\= & {} \left\{ \begin{array}{c} \left\{ 0.2\wedge 0.5,0.5\wedge 0.7,0\wedge 0.7\right\} \wedge \\ \left\{ 0.7\wedge 0.1,0.9\wedge 0.7,0\wedge 0.8\right\} \wedge \\ \left\{ 0.2\wedge 0.1,0.3\wedge 0.5,0\wedge 0.7\right\} \wedge \\ \left\{ 0.8\wedge 0.2,0\wedge 0.6,0\wedge 0.7\right\} \end{array} \right\} \\= & {} \left\{ \left\{ 0.2,0.5\right\} \wedge \left\{ 0.1,0.7\right\} \wedge \left\{ 0.2,0.3\right\} \wedge \left\{ 0.2\right\} \right\} , \\= & {} \left\{ 0.2\right\} . \end{aligned}$$

By routine calculations, we get

$$\begin{aligned} \begin{array}{ccc} \psi _{h_{\overline{\mathcal {Y} }(\mathcal {B} )}}(\mu _{2})=\left\{ 0.1\right\} ,&\psi _{h_{\overline{\mathcal {Y} }(\mathcal {B} )}}(\mu _{3})=\left\{ 0.1,0.2\right\} ,&\psi _{h_{\overline{\mathcal {Y} }(\mathcal {B} )}}(\mu _{4})=\left\{ 0.1,0.5\right\} . \end{array} \end{aligned}$$

Further

$$\begin{aligned} \eth _{h_{\underline{\mathcal {Y} }(\mathcal {B} )}}(\mu _{1})= & {} \underset{k\in \Im }{\bigwedge }\left[ \eth _{h_{\mathcal {Y} }}(\mu ,c)\bigwedge \eth _{h_{\mathcal {B} }}(k)\right] \\= & {} \left\{ \begin{array}{c} \left\{ 0.1\wedge 0.2,0.3\wedge 0.3,0.4\wedge 0.4\right\} \wedge \\ \left\{ 0.2\wedge 0.2,0.3\wedge 0.3,0\wedge 0.7\right\} \wedge \\ \left\{ 0.2\wedge 0.5,0.5\wedge 0.7,0.7\wedge 0.8\right\} \wedge \\ \left\{ 0.3\wedge 0.6,0.5\wedge 0.8,0\wedge 0.9\right\} \end{array} \right\} \\= & {} \left\{ \begin{array}{c} \left\{ 0.1,0.3,0.4\right\} \wedge \left\{ 0.2,0.3\right\} \wedge \\ \left\{ 0.2,0.5,0.7\right\} \wedge \left\{ 0.3,0.5\right\} \end{array} \right\} \\= & {} \left\{ 0.1,0.3\right\} . \end{aligned}$$

By routine calculations, we get

$$\begin{aligned} \begin{array}{ccc} \eth _{h_{\underline{\mathcal {Y} }(\mathcal {B} )}}(\mu _{2})=\left\{ 0.1,0.3\right\} ,&\eth _{h_{\underline{\mathcal {Y} }(\mathcal {B} )}}(\mu _{3})=\left\{ 0.2,0.3\right\} ,&\eth _{h_{\underline{\mathcal {Y} }(\mathcal {B} )}}(\mu _{4})=\left\{ 0.2,0.3\right\} . \end{array} \end{aligned}$$

Now,

$$\begin{aligned} \psi _{h_{\underline{\mathcal {Y} }(\mathcal {B} )}}(\mu _{1})= & {} \underset{k\in \Im }{\bigvee }\left[ \psi _{h_{\mathcal {Y} }}(\mu ,c)\bigvee \psi _{h_{\mathcal {B} }}(k)\right] \\= & {} \left\{ \begin{array}{c} \left\{ 0.2\vee 0.5,0.5\vee 0.7,0.7\vee 0\right\} \vee \\ \left\{ 0.7\vee 0.2,0.9\vee 0.3,0\vee 0.7\right\} \vee \\ \left\{ 0.2\vee 0.1,0.3\vee 0.5,0\vee 0.7\right\} \vee \\ \left\{ 0.8\vee 0.2,0\vee 0.6,0\vee 0.7\right\} \end{array} \right\} \\= & {} \left\{ \begin{array}{c} \left\{ 0.5,0.7,0.7\right\} \vee \left\{ 0.7,0.9,0.7\right\} \vee \\ \left\{ 0.2,0.5,0.7\right\} \vee \left\{ 0.8,0.6,0.7\right\} \end{array} \right\} \\= & {} \left\{ 0.8,0.9,0.7\right\} . \end{aligned}$$

Continuing in the same way, we find the other values,

$$\begin{aligned} \begin{array}{ccc} \psi _{h_{\underline{\mathcal {Y} }(\mathcal {B} )}}(\mu _{2})=\left\{ 0.7,0.9\right\} ,&\psi _{h_{\underline{\mathcal {Y} }(\mathcal {B} )}}(\mu _{3})=\left\{ 0.7,0.9,0.8\right\} ,&\psi _{h_{\underline{\mathcal {Y} }(\mathcal {B} )}}(\mu _{4})=\left\{ 0.6,0.9,0.9\right\} . \end{array} \end{aligned}$$

Thus the lower and upper *q*-ROHFR approximation operators are

$$\begin{aligned} \underline{\mathcal {Y} }(\mathcal {B} )= & {} \left\{ \begin{array}{c} \left\langle \mu _{1},\left\{ 0.1,0.3\right\} ,\left\{ 0.8,0.9,0.7\right\} \right\rangle ,\left\langle \mu _{2},\left\{ 0.1,0.3\right\} ,\left\{ 0.7,0.9\right\} \right\rangle , \\ \left\langle \mu _{3},\left\{ 0.2,0.3\right\} ,\left\{ 0.7,0.9,0.8\right\} \right\rangle ,\left\langle \mu _{4},\left\{ 0.2,0.3\right\} ,\left\{ 0.6,0.9,0.9\right\} \right\rangle \end{array} \right\} ,\\ \overline{\mathcal {Y} }(\mathcal {B} )= & {} \left\{ \begin{array}{c} \left\langle \mu _{1},\left\{ 0.6,0.8,0.9\right\} ,\left\{ 0.2\right\} \right\rangle ,\left\langle \mu _{2},\left\{ 0.6,0.8,0.9\right\} ,\left\{ 0.1\right\} \right\rangle , \\ \left\langle \mu _{3},\left\{ 0.7,0.9\right\} ,\left\{ 0.1,0.2\right\} \right\rangle ,\left\langle \mu _{4},\left\{ 0.6,0.8,0.9\right\} ,\left\{ 0.1,0.5\right\} \right\rangle \end{array} \right\} . \end{aligned}$$

Hence

$$\begin{aligned} \mathcal {Y} (\mathcal {B} )= & {} (\underline{\mathcal {Y} }(\mathcal {B} ),\overline{\mathcal {Y} } (\mathcal {B} )) \\= & {} \left\{ \begin{array}{c} \left\langle \mu _{1},\left( \left\{ 0.1,0.3\right\} ,\left\{ 0.8,0.9,0.7\right\} \right) ,\left( \left\{ 0.6,0.8,0.9\right\} ,\left\{ 0.2\right\} \right) \right\rangle , \\ \left\langle \mu _{2},\left( \left\{ 0.1,0.3\right\} ,\left\{ 0.7,0.9\right\} \right) ,\left( \left\{ 0.6,0.8,0.9\right\} ,\left\{ 0.1\right\} \right) \right\rangle , \\ \left\langle \mu _{3},\left( \left\{ 0.2,0.3\right\} ,\left\{ 0.7,0.9,0.8\right\} \right) ,\left( \left\{ 0.7,0.9\right\} ,\left\{ 0.1,0.2\right\} \right) \right\rangle , \\ \left\langle \mu _{4},\left( \left\{ 0.2,0.3\right\} ,\left\{ 0.6,0.9,0.9\right\} \right) ,\left( \left\{ 0.6,0.8,0.9\right\} ,\left\{ 0.1,0.5\right\} \right) \right\rangle \end{array} \right\} . \end{aligned}$$

### Definition 2.6

(*Ref.*^34^) Let $\mathcal {Y} (\mathcal {B} _{1})=(\underline{\mathcal {Y} }(\mathcal {B} _{1}),\overline{ \mathcal {Y} }(\mathcal {B} _{1}))$ and $\mathcal {Y} (\mathcal {B} _{2})=(\underline{\mathcal {Y} } (\mathcal {B} _{2}),\overline{\mathcal {Y} }(\mathcal {B} _{2}))$ be two *q*-ROHFRSs. Then

(1): $$\mathcal {Y} (\mathcal {B} _{1})\cup$$ $$\mathcal {Y} (\mathcal {B} _{2})=\{(\underline{\mathcal {Y} } (\mathcal {B} _{1})\cup \underline{\mathcal {Y} }(\mathcal {B} _{2})),(\overline{\mathcal {Y} } (\mathcal {B} _{1})\cup \overline{\mathcal {Y} }(\mathcal {B} _{2}))\}$$
(2): $$\mathcal {Y} (\mathcal {B} _{1})\cap$$ $$\mathcal {Y} (\mathcal {B} _{2})=\{(\underline{\mathcal {Y} } (\mathcal {B} _{1})\cap \underline{\mathcal {Y} }(\mathcal {B} _{2})),(\overline{\mathcal {Y} } (\mathcal {B} _{1})\cap \overline{\mathcal {Y} }(\mathcal {B} _{2}))\}.$$

### Definition 2.7

Let $\mathcal {Y} (\mathcal {B} _{1})=(\underline{\mathcal {Y} }(\mathcal {B} _{1}),\overline{ \mathcal {Y} }(\mathcal {B} _{1}))$ and $\mathcal {Y} (\mathcal {B} _{2})=(\underline{\mathcal {Y} } (\mathcal {B} _{2}),\overline{\mathcal {Y} }(\mathcal {B} _{2}))$ be two *q*-ROHFRSs. Then

(1): $$\mathcal {Y} (\mathcal {B} _{1})\oplus$$ $$\mathcal {Y} (\mathcal {B} _{2})=\{(\underline{ \mathcal {Y} }(\mathcal {B} _{1})\oplus \underline{\mathcal {Y} }(\mathcal {B} _{2})),(\overline{ \mathcal {Y} }(\mathcal {B} _{1})\oplus \overline{\mathcal {Y} }(\mathcal {B} _{2}))\}$$
(2): $$\mathcal {Y} (\mathcal {B} _{1})\otimes$$ $$\mathcal {Y} (\mathcal {B} _{2})=\{(\underline{ \mathcal {Y} }(\mathcal {B} _{1})\otimes \underline{\mathcal {Y} }(\mathcal {B} _{2})),( \overline{\mathcal {Y} }(\mathcal {B} _{1})\otimes \overline{\mathcal {Y} }(\mathcal {B} _{2}))\}$$
(3): $$\mathcal {Y} (\mathcal {B} _{1})\subseteq$$ $$\mathcal {Y} (\mathcal {B} _{2})=\{(\underline{ \mathcal {Y} }(\mathcal {B} _{1})\subseteq \underline{\mathcal {Y} }(\mathcal {B} _{2}))$$ and $$( \overline{\mathcal {Y} }(\mathcal {B} _{1})\subseteq \overline{\mathcal {Y} }(\mathcal {B} _{2}))\}$$
(4): $$\varpi \mathcal {Y} (\mathcal {B} _{1})=(\varpi \underline{\mathcal {Y} }(\mathcal {B} _{1}),$$ $$\varpi \overline{\mathcal {Y} }(\mathcal {B} _{1}))$$ for $$\varpi \ge 1$$
(5): $$(\mathcal {Y} (\mathcal {B} _{1}))^{\varpi }=((\underline{\mathcal {Y} }(\mathcal {B} _{1}))^{\varpi },$$ $$(\overline{\mathcal {Y} }(\mathcal {B} _{1}))^{\varpi })$$ for $$\varpi \ge 1$$
(6): $\mathcal {Y} (\mathcal {B} _{1})^{c}=(\underline{\mathcal {Y} }(\mathcal {B} _{1})^{c},$ $\overline{\mathcal {Y} }(\mathcal {B} _{1})^{c})$ where $\underline{\mathcal {Y} }(\mathcal {B} _{1})^{c}$ and $\overline{\mathcal {Y} }(\mathcal {B} _{1})^{c}$ represents the complement of *q*-RFR approximation operators $\underline{\mathcal {Y} }(\mathcal {B} _{1})$ and $\overline{\mathcal {Y} }(\mathcal {B} _{1}),$that is $\underline{\mathcal {Y} } (\mathcal {B} _{1})^{c}=\left( \psi _{h_{\underline{\mathcal {Y} }(\mathcal {B} )}},\eth _{h_{\underline{\mathcal {Y} }(\mathcal {B} )}}\right) .$
(7): $$\mathcal {Y} (\mathcal {B} _{1})=$$ $$\mathcal {Y} (\mathcal {B} _{2})$$ iff $$\underline{\mathcal {Y} } (\mathcal {B} _{1})=\underline{\mathcal {Y} }(\mathcal {B} _{2})$$ and $$\overline{\mathcal {Y} } (\mathcal {B} _{1})=\overline{\mathcal {Y} }(\mathcal {B} _{2}).$$

The score function will be used to compare/rank two or more *q*-ROHFR values. The *q*-ROHFRV with higher score value will be consider greater, and the *q*-ROHFR values with smaller score will be consider smaller. If the score values are same, we will employ the accuracy function. The *q*-ROHFRV with higher accuracy will be consider greater, and the *q*-ROHFR values with smaller accuracy will be consider smaller.

### Definition 2.8

(*Ref.*^34^) The score function for *q*-ROHFRV $\mathcal {Y} (\mathcal {B} )=( \underline{\mathcal {Y} }(\mathcal {B} ),\overline{\mathcal {Y} }(\mathcal {B} ))=((\underline{ \eth },\underline{\psi }),(\overline{\eth },\overline{\psi }))$ is given as:

$$\begin{aligned} SR(\mathcal {Y} (\mathcal {B} ))=\frac{1}{4}\left( \begin{array}{c} 2+\frac{1}{M_{\mathcal {F}}}\sum \limits _{\underline{\mu _{i}}\in \eth _{h_{ \underline{\mathcal {Y} }(\mathcal {B} )}}}\left\{ \underline{\vartheta _{i}}\right\} + \frac{1}{N_{\mathcal {F}}}\sum \limits _{\overline{\vartheta _{i}}\in \eth _{h_{ \overline{\mathcal {Y} }(\mathcal {B} )}}}\left\{ \overline{\vartheta _{i}}\right\} - \\ \frac{1}{M_{\mathcal {F}}}\sum \limits _{\underline{\delta _{i}}\in \psi _{h_{ \underline{\mathcal {Y} }(\mathcal {B} )}}}(\underline{\delta _{i}})-\frac{1}{M_{\mathcal { F}}}\sum \limits _{\overline{\delta _{i}}\in \psi _{h_{\overline{\mathcal {Y} } (\mathcal {B} )}}}(\overline{\delta _{i}}) \end{array} \right) , \end{aligned}$$

The accuracy function for *q*-ROHFRV $\mathcal {Y} (\mathcal {B} )=(\underline{\mathcal {Y} } (\mathcal {B} ),\overline{\mathcal {Y} }(\mathcal {B} ))=((\underline{\wp },\underline{ \Im }),(\overline{\eth },\overline{\psi }))$ is given as;

$$\begin{aligned} \mathbf {AC}\mathcal {Y} (\mathcal {B} )=\frac{1}{4}\left( \begin{array}{c} \frac{1}{M_{\mathcal {F}}}\sum \limits _{\vartheta _{i}\in \eth _{h_{\overline{ \mathcal {Y} }(\mathcal {B} )}}}(\overline{\vartheta _{i}})+\frac{1}{M_{\mathcal {F}}} \sum \limits _{\vartheta _{i}\in \eth _{h_{\overline{\mathcal {Y} }(\mathcal {B} )}}}( \overline{\vartheta _{i}})+ \\ \frac{1}{M_{\mathcal {F}}}\sum \limits _{\underline{\delta _{i}}\in \psi _{h_{ \underline{\mathcal {Y} }(\mathcal {B} )}}}(\underline{\delta _{i}})+\frac{1}{M_{\mathcal { F}}}\sum \limits _{\overline{\delta _{i}}\in \psi _{h_{\overline{\mathcal {Y} } (\mathcal {B} )}}}(\overline{\delta _{i}}) \end{array} \right) , \end{aligned}$$

where $M_{\mathcal {F}}$ and $N_{\mathcal {F}}$ represent the number of elements in $\eth _{h_{g}}$ and $\psi _{h_{g}}$ respectively.

### Definition 2.9

(*Ref.*^34^) Suppose $\mathcal {Y} (\mathcal {B} _{1})=(\underline{\mathcal {Y} }(\mathcal {B} _{1}), \overline{\mathcal {Y} }(\mathcal {B} _{1}))$ and $\mathcal {Y} (\mathcal {B} _{2})=(\underline{ \mathcal {Y} }(\mathcal {B} _{2}),\overline{\mathcal {Y} }(\mathcal {B} _{2}))$ are two *q*-ROHFR values. Then

(1): If $$SR(\mathcal {Y} (\mathcal {B} _{1}))>SR(\mathcal {Y} (\mathcal {B} _{2})),$$ then $$\mathcal {Y} (\mathcal {B} _{1})>\mathcal {Y} (\mathcal {B} _{2}),$$
(2): If $$SR(\mathcal {Y} (\mathcal {B} _{1}))\prec SR(\mathcal {Y} (\mathcal {B} _{2})),$$ then $$\mathcal {Y} (\mathcal {B} _{1})\prec \mathcal {Y} (\mathcal {B} _{2}),$$
(3): If $$SR(\mathcal {Y} (\mathcal {B} _{1}))=SR(\mathcal {Y} (\mathcal {B} _{2})),$$ then
  (a): If $$\mathbf {AC}\mathcal {Y} (\mathcal {B} _{1})>\mathbf {AC}\mathcal {Y} (\mathcal {B} _{2})$$ then $$\mathcal {Y} (\mathcal {B} _{1})>\mathcal {Y} (\mathcal {B} _{2}),$$
  (b): If $$\mathbf {AC}\mathcal {Y} (\mathcal {B} _{1})\prec \mathbf {AC}\mathcal {Y} (\mathcal {B} _{2})$$ then $$\mathcal {Y} (\mathcal {B} _{1})\prec \mathcal {Y} (\mathcal {B} _{2}),$$
  (c): If $$\mathbf {AC}\mathcal {Y} (\mathcal {B} _{1})=\mathbf {AC}\mathcal {Y} (\mathcal {B} _{2})$$ then $$\mathcal {Y} (\mathcal {B} _{1})=\mathcal {Y} (\mathcal {B} _{2}).$$

## The *q*-rung orthopair hesitant fuzzy rough aggregation operators

In this part, we provide a novel concept of *q*-ROHF rough AOPs by incorporating RS and *q*-ROHFR aggregation operators to get aggregation concepts of *q*-ROHFREWG, *q*-ROHFREOWG, and *q*-ROHFREHWG operators. In addition, some of the essential features of the concepts are examined.

### *q*-rung orthopair hesitant fuzzy rough Einstein weighted geometric aggregation operator

This section describes the *q*-ROHFREWG aggregation operator and highlights its important features.

#### Definition 3.1

Let $\mathcal {Y} (\varrho _{t})=(\underline{\mathcal {Y} }(\varrho _{t}),\overline{\mathcal {Y} }(\varrho _{t}))$ $(t=1,2,3,4,\ldots ,n)$ be the collection of *q*-ROHFR values. Then *q*-ROHFREWG operator is follows as:

$$\begin{aligned} q-ROHFREWG\left( \mathcal {Y} (\varrho _{1}),\mathcal {Y} (\varrho _{2}),\ldots ,\mathcal {Y} (\varrho _{n})\right) =\left( \bigoplus \limits _{t=1}^{n}\left( \underline{\mathcal {Y} } (\varrho _{t})\right) ^{\varpi _{t}},\bigoplus \limits _{t=1}^{n}\left( \overline{\mathcal {Y} }(\varrho _{t})\right) ^{\varpi _{t}}\right) . \end{aligned}$$

where $\varpi =\left( \varpi _{1},\varpi _{2},\ldots ,\varpi _{n}\right) ^{T}$ is the weight vector such that $\bigoplus \nolimits _{t=1}^{n}\varpi _{t}=1$ and $0\le$ $\varpi _{t}\le 1.$

#### Theorem 3.2

*Let* $\mathcal {Y} (\varrho _{t})=(\underline{\mathcal {Y} }(\varrho _{t}),\overline{ \mathcal {Y} }(\varrho _{t}))$ $(t=1,2,3,\ldots ,n)$ *be the collection of* *q-ROHFR values with weight vectors* $\varpi =\left( \varpi _{1},\varpi _{2},\ldots ,\varpi _{n}\right) ^{T}$ *such that* $\bigoplus \nolimits _{t=1}^{n}\varpi _{t}=1$ *and* $0\le$ $\varpi _{t}\le 1.$ *Then* *q-ROHFREWG operator is described as:*

$$\begin{aligned}&q-ROHFREWG\left( \mathcal {Y} (\varrho _{1}),\mathcal {Y} (\varrho _{2}),\ldots \mathcal {Y} (\varrho _{n})\right) \\&\quad =\left( \overset{n}{\underset{t=1}{\otimes }}\left( \underline{ \mathcal {Y} }(\varrho _{t})\right) ^{\varpi _{t}},\overset{n}{\underset{t=1}{\otimes }} \left( \overline{\mathcal {Y} }(\varrho _{t})\right) ^{\varpi _{t}}\right) \\&\quad =\left( \begin{array}{c} \text { }\left\{ \bigcup \limits _{\underline{\vartheta _{h_{t}}}\in \eth _{h_{ \underline{\mathcal {Y} }(\varrho )}}}\left( \frac{\sqrt[q] {2\overset{n}{\underset{t=1}{ \otimes }}\left( \underline{\vartheta _{h_{t}}}^{q}\right) ^{\varpi _{t}}}}{ \sqrt[q] {\overset{n}{\underset{t=1}{\otimes }}\left( 2-\underline{\vartheta _{h_{t}}}^{q}\right) ^{\varpi _{t}}+\overset{n}{\underset{t=1}{\otimes }} \left( \underline{\vartheta _{h_{t}}}^{q}\right) ^{\varpi _{t}}}}\right) ,\bigcup \limits _{\underline{\delta _{h_{t}}}\in \psi _{h_{\underline{\mathcal {Y} } (\varrho )}}}\left( \frac{\sqrt[q] {\overset{n}{\underset{t=1}{\otimes }}\left( 1+\underline{\delta _{h_{t}}^{q}}\right) ^{\varpi _{t}}-\overset{n}{\underset{ t=1}{\otimes }}\left( 1-\underline{\delta _{h_{t}}^{q}}\right) ^{\varpi _{t}}}}{ \sqrt[q] {\overset{n}{\underset{t=1}{\otimes }}\left( 1+\underline{\delta _{h_{t}}^{q}}\right) ^{\varpi _{t}}+\overset{n}{\underset{t=1}{\otimes }} \left( 1-\underline{\delta _{h_{t}}^{q}}\right) ^{\varpi _{t}}}}\right) \right\} \\ \left\{ \bigcup \limits _{\overline{\vartheta _{h_{t}}}\in \psi _{h_{\overline{ \mathcal {Y} }(\varrho )}}}\left( \frac{\sqrt[q] {2\overset{n}{\underset{t=1}{\otimes }} \left( \overline{\vartheta _{h_{t}}^{q}}\right) ^{\varpi _{t}}}}{\sqrt[q] { \overset{n}{\underset{t=1}{\otimes }}\left( 2-\overline{\vartheta _{h_{t}}^{q}} \right) ^{\varpi _{t}}+\overset{n}{\underset{t=1}{\otimes }}\left( \overline{ \vartheta _{h_{t}}^{q}}\right) ^{\varpi _{t}}}}\right) ,\bigcup \limits _{ \overline{\delta _{h_{t}}}\in \psi _{h_{\overline{\mathcal {Y} }(\varrho )}}}\left( \frac{ \sqrt[q] {\overset{n}{\underset{t=1}{\otimes }}\left( 1+\overline{\delta _{h_{t}}^{q}}\right) ^{\varpi _{t}}-\overset{n}{\underset{t=1}{\otimes }} \left( 1-\overline{\delta _{h_{t}}^{q}}\right) ^{\varpi _{t}}}}{\sqrt[q] { \overset{n}{\underset{t=1}{\otimes }}\left( 1+\overline{\delta _{h_{t}}^{q}} \right) ^{\varpi _{t}}+\overset{n}{\underset{t=1}{\otimes }}\left( 1- \overline{\delta _{h_{t}}^{q}}\right) ^{\varpi _{t}}}}\right) \right\} \end{array} \right) , \end{aligned}$$

*where* $\varpi =\left( \varpi _{1},\varpi _{2},\ldots ,\varpi _{n}\right) ^{T}$ *is weight vector such that* $\oplus _{t=1}^{n}\varpi _{t}=1$ *and* $0\le$ $\varpi _{t}\le 1.$

#### Proof

We ¼ induction. If $n=2$, then

$$\begin{aligned}\left( \mathcal {Y} (\varrho _{1})\otimes \mathcal {Y} (\varrho _{2})\right) &\quad =\left( \underline{ \mathcal {Y} }(\varrho _{1})\otimes \underline{\mathcal {Y} }(\varrho _{2}),\overline{\mathcal {Y} }(\varrho _{1})\otimes \overline{\mathcal {Y} }(\varrho _{2})\right) \\&\qquad q-ROHFREWG\left( \mathcal {Y} (\varrho _{1}),\mathcal {Y} (\varrho _{2})\right) =\left( \overset{ 2}{\underset{t=1}{\otimes }}\left( \underline{\mathcal {Y} }(\varrho _{t})\right) ^{\varpi _{t}},\overset{2}{\underset{t=1}{\otimes }}\left( \overline{\mathcal {Y} } (\varrho _{t})\right) ^{\varpi _{t}}\right) \\&\quad =\left( \begin{array}{c} \text { }\left\{ \bigcup \limits _{\underline{\vartheta _{h_{t}}}\in \eth _{h_{ \underline{\mathcal {Y} }(\varrho )}}}\left( \frac{\sqrt[q] {2\overset{2}{\underset{t=1}{ \otimes }}\left( \underline{\vartheta _{h_{t}}}^{q}\right) ^{\varpi _{t}}}}{ \sqrt[q] {\overset{2}{\underset{t=1}{\otimes }}\left( 2-\underline{\vartheta _{h_{t}}}^{q}\right) ^{\varpi _{t}}+\overset{2}{\underset{t=1}{\otimes }} \left( \underline{\vartheta _{h_{t}}}^{q}\right) ^{\varpi _{t}}}}\right) ,\bigcup \limits _{\underline{\delta _{h_{t}}}\in \psi _{h_{\underline{\mathcal {Y} } (\varrho )}}}\left( \frac{\sqrt[q] {\overset{2}{\underset{t=1}{\otimes }}\left( 1+\underline{\delta _{h_{t}}^{q}}\right) ^{\varpi _{t}}-\overset{2}{\underset{ t=1}{\otimes }}\left( 1-\underline{\delta _{h_{t}}^{q}}\right) ^{\varpi _{t}}}}{ \sqrt[q] {\overset{2}{\underset{t=1}{\otimes }}\left( 1+\underline{\delta _{h_{t}}^{q}}\right) ^{\varpi _{t}}+\overset{2}{\underset{t=1}{\otimes }} \left( 1-\underline{\delta _{h_{t}}^{q}}\right) ^{\varpi _{t}}}}\right) \right\} , \\ \left\{ \bigcup \limits _{\overline{\vartheta _{h_{t}}}\in \psi _{h_{\overline{ \mathcal {Y} }(\varrho )}}}\left( \frac{\sqrt[q] {2\overset{2}{\underset{t=1}{\otimes }} \left( \overline{\vartheta _{h_{t}}^{q}}\right) ^{\varpi _{t}}}}{\sqrt[q] { \overset{2}{\underset{t=1}{\otimes }}\left( 2-\overline{\vartheta _{h_{t}}^{q}} \right) ^{\varpi _{t}}+\overset{2}{\underset{t=1}{\otimes }}\left( \overline{ \vartheta _{h_{t}}^{q}}\right) ^{\varpi _{t}}}}\right) ,\bigcup \limits _{ \overline{\delta _{h_{t}}}\in \psi _{h_{\overline{\mathcal {Y} }(\varrho )}}}\left( \frac{ \sqrt[q] {\overset{2}{\underset{t=1}{\otimes }}\left( 1+\overline{\delta _{h_{t}}^{q}}\right) ^{\varpi _{t}}-\overset{2}{\underset{t=1}{\otimes }} \left( 1-\overline{\delta _{h_{t}}^{q}}\right) ^{\varpi _{t}}}}{\sqrt[q] { \overset{2}{\underset{t=1}{\otimes }}\left( 1+\overline{\delta _{h_{t}}^{q}} \right) ^{\varpi _{t}}+\overset{2}{\underset{t=1}{\otimes }}\left( 1- \overline{\delta _{h_{t}}^{q}}\right) ^{\varpi _{t}}}}\right) \right\} \end{array} \right) , \end{aligned}$$

hence the result holds true for $n=2$. Assume it is valid for $n=k$:

$$\begin{aligned}&q-ROHFREWG\left( \mathcal {Y} (\varrho _{1}),\mathcal {Y} (\varrho _{2})\ldots \mathcal {Y} (\varrho _{k})\right) =\left( \overset{k}{\underset{t=1}{\otimes }}\left( \underline{ \mathcal {Y} }(\varrho _{t})\right) ^{\varpi _{t}},\overset{k}{\underset{t=1}{\otimes }} \left( \overline{\mathcal {Y} }(\varrho _{t})\right) ^{\varpi _{t}}\right) \\&\quad =\left( \begin{array}{c} \text { }\left\{ \bigcup \limits _{\underline{\vartheta _{h_{t}}}\in \eth _{h_{ \underline{\mathcal {Y} }(\varrho )}}}\left( \frac{\sqrt[q] {2\overset{k}{\underset{t=1}{ \otimes }}\left( \underline{\vartheta _{h_{t}}}^{q}\right) ^{\varpi _{t}}}}{ \sqrt[q] {\overset{k}{\underset{t=1}{\otimes }}\left( 2-\underline{\vartheta _{h_{t}}}^{q}\right) ^{\varpi _{t}}+\overset{k}{\underset{t=1}{\otimes }} \left( \underline{\vartheta _{h_{t}}}^{q}\right) ^{\varpi _{t}}}}\right) ,\bigcup \limits _{\underline{\delta _{h_{t}}}\in \psi _{h_{\underline{\mathcal {Y} } (\varrho )}}}\left( \frac{\sqrt[q] {\overset{k}{\underset{t=1}{\otimes }}\left( 1+\underline{\delta _{h_{t}}^{q}}\right) ^{\varpi _{t}}-\overset{k}{\underset{ t=1}{\otimes }}\left( 1-\underline{\delta _{h_{t}}^{q}}\right) ^{\varpi _{t}}}}{ \sqrt[q] {\overset{k}{\underset{t=1}{\otimes }}\left( 1+\underline{\delta _{h_{t}}^{q}}\right) ^{\varpi _{t}}+\overset{k}{\underset{t=1}{\otimes }} \left( 1-\underline{\delta _{h_{t}}^{q}}\right) ^{\varpi _{t}}}}\right) \right\} , \\ \left\{ \bigcup \limits _{\overline{\vartheta _{h_{t}}}\in \psi _{h_{\overline{ \mathcal {Y} }(\varrho )}}}\left( \frac{\sqrt[q] {2\overset{k}{\underset{t=1}{\otimes }} \left( \overline{\vartheta _{h_{t}}^{q}}\right) ^{\varpi _{t}}}}{\sqrt[q] { \overset{k}{\underset{t=1}{\otimes }}\left( 2-\overline{\vartheta _{h_{t}}^{q}} \right) ^{\varpi _{t}}+\overset{k}{\underset{t=1}{\otimes }}\left( \overline{ \vartheta _{h_{t}}^{q}}\right) ^{\varpi _{t}}}}\right) ,\bigcup \limits _{ \overline{\delta _{h_{t}}}\in \psi _{h_{\overline{\mathcal {Y} }(\varrho )}}}\left( \frac{ \sqrt[q] {\overset{k}{\underset{t=1}{\otimes }}\left( 1+\overline{\delta _{h_{t}}^{q}}\right) ^{\varpi _{t}}-\overset{k}{\underset{t=1}{\otimes }} \left( 1-\overline{\delta _{h_{t}}^{q}}\right) ^{\varpi _{t}}}}{\sqrt[q] { \overset{k}{\underset{t=1}{\otimes }}\left( 1+\overline{\delta _{h_{t}}^{q}} \right) ^{\varpi _{t}}+\overset{k}{\underset{t=1}{\otimes }}\left( 1- \overline{\delta _{h_{t}}^{q}}\right) ^{\varpi _{t}}}}\right) \right\} \end{array} \right) . \end{aligned}$$

Further, we show that the result hold for $n=k+1.$ Consider

$$\begin{aligned}&q-ROHFREWG\left( \mathcal {Y} (\varrho _{1}),\mathcal {Y} (\varrho _{2}),\ldots ,\mathcal {Y} (\varrho _{k}),\mathcal {Y} (\varrho _{k+1})\right) \\&\quad =\left( \overset{k}{\underset{t=1}{\otimes }}\left( \left( \underline{\mathcal {Y} } (\varrho _{t})\right) ^{\varpi _{t}}\right) \otimes \left( \underline{\mathcal {Y} } (\varrho _{k+1})\right) ^{w_{k+1}},\overset{k}{\underset{t=1}{\otimes }}\left( \left( \overline{\mathcal {Y} }(\varrho _{t})\right) ^{\varpi _{t}}\right) \otimes \left( \overline{\mathcal {Y} }(\varrho _{k+1})\right) ^{w_{k+1}}\right) \\&\quad =\left( \begin{array}{c} \text { }\left\{ \bigcup \limits _{\underline{\vartheta _{h_{t}}}\in \eth _{h_{ \underline{\mathcal {Y} }(\varrho )}}}\left( \frac{\sqrt[q] {2\overset{k+1}{\underset{t=1 }{\otimes }}\left( \underline{\vartheta _{h_{t}}}^{q}\right) ^{\varpi _{t}}}}{ \sqrt[q] {\overset{k+1}{\underset{t=1}{\otimes }}\left( 2-\underline{\vartheta _{h_{t}}^{q}}\right) ^{\varpi _{t}}+\overset{k+1}{\underset{t=1}{\otimes }} \left( \underline{\vartheta _{h_{t}}}^{qq}\right) ^{\varpi _{t}}}}\right) ,\bigcup \limits _{\underline{\delta _{h_{t}}}\in \psi _{h_{\underline{\mathcal {Y} } (\varrho )}}}\left( \frac{\sqrt[q] {\overset{k+1}{\underset{t=1}{\otimes }} \left( 1+\underline{\delta _{h_{t}}^{q}}\right) ^{\varpi _{t}}-\overset{k+1}{ \underset{t=1}{\otimes }}\left( 1-\underline{\delta _{h_{t}}^{q}}\right) ^{\varpi _{t}}}}{\sqrt[q] {\overset{k+1}{\underset{t=1}{\otimes }}\left( 1+ \underline{\delta _{h_{t}}^{q}}\right) ^{\varpi _{t}}+\overset{k+1}{\underset{ t=1}{\otimes }}\left( 1-\underline{\delta _{h_{t}}^{q}}\right) ^{\varpi _{t}}}} \right) \right\} , \\ \left\{ \bigcup \limits _{\overline{\vartheta _{h_{t}}}\in \psi _{h_{\overline{ \mathcal {Y} }(\varrho )}}}\left( \frac{\sqrt[q] {2\overset{k+1}{\underset{t=1}{\otimes } }\left( \overline{\vartheta _{h_{t}}^{q}}\right) ^{\varpi _{t}}}}{\sqrt[q] { \overset{k+1}{\underset{t=1}{\otimes }}\left( 2-\overline{\vartheta _{h_{t}}^{q} }\right) ^{\varpi _{t}}+\overset{k+1}{\underset{t=1}{\otimes }}\left( \overline{\vartheta _{h_{t}}^{q}}\right) ^{\varpi _{t}}}}\right) ,\bigcup \limits _{\overline{\delta _{h_{t}}}\in \psi _{h_{\overline{\mathcal {Y} }(\varrho )}}}\left( \frac{\sqrt[q] {\overset{k}{\underset{t=1}{\otimes }}\left( 1+ \overline{\delta _{h_{t}}^{q}}\right) ^{\varpi _{t}}-\overset{k+1}{\underset{t=1 }{\otimes }}\left( 1-\overline{\delta _{h_{t}}^{q}}\right) ^{\varpi _{t}}}}{ \sqrt[q] {\overset{k+1}{\underset{t=1}{\otimes }}\left( 1+\overline{\delta _{h_{t}}^{q}}\right) ^{\varpi _{t}}+\overset{k+1}{\underset{t=1}{\otimes }} \left( 1-\overline{\delta _{h_{t}}^{q}}\right) ^{\varpi _{t}}}}\right) \right\} \end{array} \right) . \end{aligned}$$

Hence the result hold for $n=k+1.$ Therefore, the result is true for all $n\ge 1.$ $\square$

#### Theorem 3.3

*Let* $\mathcal {Y} (\varrho _{t})=(\underline{\mathcal {Y} }(\varrho _{t}),\overline{ \mathcal {Y} }(\varrho _{t}))$ $(t=1,2,3,\ldots ,n)$ *be the collection of* *q*-ROHFRVs *and* $\varpi =\left( \varpi _{1},\varpi _{2},\ldots ,\varpi _{n}\right) ^{T}$ *is weight vector such that* $\varpi _{t}\in [0,1]$ *and* $\oplus _{t=1}^{n}\varpi _{t}=1.$ *Then* *q-ROHFREWG operator satisfy the following properties:*

(1): **Idempotency:** *If* $\mathcal {Y} (\varrho _{t})=\mathfrak {F} (\varrho )$ *for* $t=1,2,3,\ldots ,n$ *where* $\mathfrak {F} (\varrho )=\left( \underline{\mathfrak {F} } (\varrho ),\overline{\mathfrak {F} }(\varrho )\right) =\left( (\underline{b_{h(x)}}, \underline{d_{h(x)}}),(\overline{b}_{h(x)},\overline{d_{h(x)}})\right) .$ *Then*
  $$\begin{aligned} q-ROHFREWG\left( \mathcal {Y} (\varrho _{1}),\mathcal {Y} (\varrho _{2}),\ldots ,\mathcal {Y} (\varrho _{n})\right) =\mathfrak {F} (\varrho ). \end{aligned}$$
(2): **Boundedness:** *Let* $\left( \mathcal {Y} (\varrho )\right) _{\min }=\left( \underset{t}{\min }\underline{\mathcal {Y} }\left( \varrho _{t}\right) ,\underset{t}{ \max }\overline{\mathcal {Y} }(\varrho _{t})\right)$ and $\left( \mathcal {Y} (\varrho )\right) _{\max }=$ $\left( \underset{t}{\max }\underline{\mathcal {Y} }\left( \varrho _{t}\right) ,\underset{t}{\min }\overline{\mathcal {Y} }(\varrho _{t})\right) .$ *Then*
  $$\begin{aligned} \left( \mathcal {Y} (\varrho )\right) _{\min }\le q-ROHFREWG\left( \mathcal {Y} (\varrho _{1}),\mathcal {Y} (\varrho _{2}),\ldots ,\mathcal {Y} (\varrho _{n})\right) \le \left( \mathcal {Y} (\varrho )\right) _{\max }. \end{aligned}$$
(3): **Monotonicity: ***Suppose* $\mathfrak {F} (\varrho )=\left( \underline{\mathfrak {F} }(\varrho _{t}),\overline{\mathfrak {F} }(\varrho _{t})\right) (t=1,2,3,\ldots ,n)$ *is another collection of* *q-ROHFR values such that* $\underline{\mathfrak {F} }(\varrho _{t})\le \underline{\mathcal {Y} }\left( \varrho _{t}\right)$ and $\overline{\mathfrak {F} }(\varrho _{t})\le \overline{\mathcal {Y} } (\varrho _{t})$. *Then*

$$\begin{aligned} q-ROHFREWG\left( \mathfrak {F} (\varrho _{1}),\mathfrak {F} (\varrho _{2}),\ldots ,\mathfrak {F} (\varrho _{n})\right) \le q-ROHFREWG\left( \mathcal {Y} (\varrho _{1}),\mathcal {Y} (\varrho _{2}),\ldots ,\mathcal {Y} (\varrho _{n})\right) . \end{aligned}$$

#### Proof

(1): **Idempotency:** As $\mathcal {Y} (\varrho _{t})=\mathfrak {F} (\varrho )$ (for all $t=1,2,3,\ldots ,n$) where $\mathfrak {F} (\varrho _{t})=\left( \underline{ \mathfrak {F} }(\varrho ),\overline{\mathfrak {F} }(\varrho )\right) =\left( ( \underline{b_{h(x)}},\underline{d_{h(x)}}),(\overline{b_{h(x)}},\overline{ d_{h(x)}})\right) .$ It follows that
  $$\begin{aligned}&q-ROHFREWG\left( \mathcal {Y} (\varrho _{1}),\mathcal {Y} (\varrho _{2}),\ldots ,\mathcal {Y} (\varrho _{n})\right) =\left( \overset{n}{\underset{t=1}{\otimes }}\left( \underline{ \mathcal {Y} }(\varrho _{t})\right) ^{\varpi _{t}},\overset{n}{\underset{t=1}{\otimes }} \left( \overline{\mathcal {Y} }(\varrho _{t})\right) ^{\varpi _{t}}\right) \\&\quad =\left( \begin{array}{c} \text { }\left\{ \bigcup \limits _{\underline{\vartheta _{h_{t}}}\in \eth _{h_{ \underline{\mathcal {Y} }(\varrho )}}}\left( \frac{\sqrt[q] {2\overset{n}{\underset{t=1}{ \otimes }}\left( \underline{\vartheta _{h_{t}}}^{q}\right) ^{\varpi _{t}}}}{ \sqrt[q] {\overset{n}{\underset{t=1}{\otimes }}\left( 2-\underline{\vartheta _{h_{t}}}^{q}\right) ^{\varpi _{t}}+\overset{n}{\underset{t=1}{\otimes }} \left( \underline{\vartheta _{h_{t}}}\right) ^{\varpi _{t}}}}\right) ,\bigcup \limits _{\underline{\delta _{h_{t}}}\in \psi _{h_{\underline{\mathcal {Y} } (\varrho )}}}\left( \frac{\sqrt[q] {\overset{n}{\underset{t=1}{\otimes }}\left( 1+\underline{\delta _{h_{t}}^{q}}\right) ^{\varpi _{t}}-\overset{n}{\underset{ t=1}{\otimes }}\left( 1-\underline{\delta _{h_{t}}^{q}}\right) ^{\varpi _{t}}}}{ \sqrt[q] {\overset{n}{\underset{t=1}{\otimes }}\left( 1+\underline{\delta _{h_{t}}^{q}}\right) ^{\varpi _{t}}+\overset{n}{\underset{t=1}{\otimes }} \left( 1-\underline{\delta _{h_{t}}^{q}}\right) ^{\varpi _{t}}}}\right) \right\} \\ \left\{ \bigcup \limits _{\overline{\vartheta _{h_{t}}}\in \psi _{h_{\overline{ \mathcal {Y} }(\varrho )}}}\left( \frac{\sqrt[q] {2\overset{n}{\underset{t=1}{\otimes }} \left( \overline{\vartheta _{h_{t}}^{q}}\right) ^{\varpi _{t}}}}{\sqrt[q] { \overset{n}{\underset{t=1}{\otimes }}\left( 2-\overline{\vartheta _{h_{t}}^{q}} \right) ^{\varpi _{t}}+\overset{n}{\underset{t=1}{\otimes }}\left( \overline{ \vartheta _{h_{t}}^{q}}\right) ^{\varpi _{t}}}}\right) ,\bigcup \limits _{ \overline{\delta _{h_{t}}}\in \psi _{h_{\overline{\mathcal {Y} }(\varrho )}}}\left( \frac{ \sqrt[q] {\overset{n}{\underset{t=1}{\otimes }}\left( 1+\overline{\delta _{h_{t}}^{q}}\right) ^{\varpi _{t}}-\overset{n}{\underset{t=1}{\otimes }} \left( 1-\overline{\delta _{h_{t}}^{q}}\right) ^{\varpi _{t}}}}{\sqrt[q] { \overset{n}{\underset{t=1}{\otimes }}\left( 1+\overline{\delta _{h_{t}}^{q}} \right) ^{\varpi _{t}}+\overset{n}{\underset{t=1}{\otimes }}\left( 1- \overline{\delta _{h_{t}}^{q}}\right) ^{\varpi _{t}}}}\right) \right\} \end{array} \right) , \end{aligned}$$
  for all *t*,  $\mathcal {Y} (\varrho _{t})=\mathfrak {F} (\varrho )=$ $\left( \underline{ \mathfrak {F} }(\varrho ),\overline{\mathfrak {F} }(\varrho )\right) =\left( ( \underline{b_{h(x)}},\underline{d_{h(x)}}),(\overline{b_{h(x)}},\overline{ d_{h(x)}})\right) .$ Therefore,
  $$\begin{aligned}= & {} \left[ \begin{array}{c} \left( \begin{array}{c} \bigcup \limits _{\underline{b_{h(x)}}\in \eth _{h_{\underline{\mathcal {Y} }(\varrho )}}}\left( \frac{\sqrt[q] {2\overset{n}{\underset{t=1}{\otimes }}\left( \left( \underline{b_{h(x)}}\right) ^{q}\right) ^{^{\varpi _{t}}}}}{\sqrt[q] { \overset{n}{\underset{t=1}{\otimes }}\left( 2-\left( \underline{b_{h(x)}} \right) ^{q}\right) ^{^{\varpi _{t}}}+\overset{n}{\underset{t=1}{\otimes }} \left( \left( \underline{b_{h(x)}}\right) ^{q}\right) ^{^{\varpi _{t}}}}} \right) , \\ \text { }\bigcup \limits _{\underline{d_{h(x)}}\in \psi _{h_{\underline{\mathcal {Y} } (\varrho )}}}\left( \frac{\sqrt[q] {\overset{n}{\underset{t=1}{\otimes }}\left( 1+\left( \underline{d_{h(x)}}\right) ^{q}\right) ^{^{\varpi _{t}}}-\overset{n }{\underset{t=1}{\otimes }}\left( 1-\left( \underline{d_{h(x)}}\right) ^{q}\right) ^{^{\varpi _{t}}}}}{\sqrt[q] {\overset{n}{\underset{t=1}{\otimes } }\left( 1+\left( \underline{d_{h(x)}}\right) ^{q}\right) ^{^{\varpi _{t}}}+ \overset{n}{\underset{t=1}{\otimes }}\left( 1-\left( \underline{d_{h(x)}} \right) ^{q}\right) ^{^{\varpi _{t}}}}}\right) \end{array} \right) \\ \left( \begin{array}{c} \bigcup \limits _{\overline{b_{h(x)}}\in \eth _{h_{\overline{\mathcal {Y} }(\varrho )}}}\left( \frac{\sqrt[q] {2\overset{n}{\underset{t=1}{\otimes }}\left( \left( \overline{b_{h(x)}}\right) ^{q}\right) ^{^{\varpi _{t}}}}}{\sqrt[q] { \overset{n}{\underset{t=1}{\otimes }}\left( 2-\left( \overline{b_{h(x)}} \right) ^{q}\right) ^{^{\varpi _{t}}}+\overset{n}{\underset{t=1}{\otimes }} \left( \left( \overline{b_{h(x)}}\right) ^{q}\right) ^{^{\varpi _{t}}}}} \right) , \\ \text { }\bigcup \limits _{\overline{b_{h(x)}}\in \psi _{h_{\overline{\mathcal {Y} } (\varrho )}}}\left( \frac{\sqrt[q] {\overset{n}{\underset{t=1}{\otimes }}\left( 1+\left( \overline{d_{h(x)}}\right) ^{q}\right) ^{^{\varpi _{t}}}-\overset{n}{\underset{t=1}{\otimes }}\left( 1-\left( \overline{d_{h(x)}}\right) ^{q}\right) ^{^{\varpi _{t}}}}}{\sqrt[q] {\overset{n}{\underset{t=1}{\otimes } }\left( 1+\left( \overline{d_{h(x)}}\right) ^{q}\right) ^{^{\varpi _{t}}}+ \overset{n}{\underset{t=1}{\otimes }}\left( 1-\left( \overline{d_{h(x)}} \right) ^{q}\right) ^{^{\varpi _{t}}}}}\right) \end{array} \right) \end{array} \right] \\= & {} \left[ \left( 1-\left( 1-\underline{b_{h(x)}}\right) ,\underline{d_{h(x)}} \right) , \left( 1-\left( 1-\overline{b}_{h(x)}\right) ,\overline{d} _{h(x)}\right) \right] \\= & {} \left( \underline{\mathfrak {F} }(\varrho ),\overline{\mathfrak {F} }(\varrho )\right) =\mathfrak {F} (\varrho ). \end{aligned}$$
  Hence $$q-ROHFREWG\left( \mathcal {Y} (\varrho _{1}),\mathcal {Y} (\varrho _{2}),\ldots ,\mathcal {Y} (\varrho _{n})\right) =\mathfrak {F} (\varrho ).$$
(2): **Boundedness:** As
  $$\begin{aligned} \left( \underline{\mathcal {Y} }\left( \varrho \right) \right) ^{-}= & {} \left[ \begin{array}{c} \left( \underset{t}{\min }\left\{ \underline{\vartheta _{h_{t}}}\right\} , \underset{t}{\max }\left\{ \underline{\delta _{h_{t}}}\right\} \right) , \\ \left( \underset{t}{\min }\{\overline{\vartheta _{h_{t}}}\},\underset{t}{\max } \left\{ \overline{\delta _{h}}_{_{t}}\right\} \right) \end{array} \right] \\ \left( \underline{\mathcal {Y} }\left( \varrho \right) \right) ^{+}= & {} \left[ \begin{array}{c} \left( \underset{t}{\max }\{\underline{\vartheta _{h_{t}}}\},\underset{t}{\min } \left\{ \underline{\delta _{h_{t}}}\right\} \right) , \\ \left( \underset{t}{\max }\{\overline{\vartheta _{h_{t}}}\},\underset{t}{\min } \left\{ \overline{\delta _{h}}_{_{t}}\right\} \right) \end{array} \right] \end{aligned}$$
  and $\mathcal {Y} (\varrho _{t})=\left[ \left( \underline{\eth _{t}},\underline{\psi }_{t} \right) ,\left( \overline{\eth _{t}},\overline{\psi }_{t}\right) \right] .$ To prove that
  $$\begin{aligned} \left( \mathcal {Y} (\varrho )\right) ^{-}\le q-ROHFREWG\left( \mathcal {Y} (\varrho _{1}),\mathcal {Y} (\varrho _{2}),\ldots ,\mathcal {Y} (\varrho _{n})\right) \le \left( \mathcal {Y} (\varrho )\right) ^{+}. \end{aligned}$$
  Let $g(\varkappa )$=$\sqrt[3] {\frac{2-\varkappa ^{3}}{\varkappa ^{3}}} ,\varkappa \in (0,1],$ then $g^{\prime }(\varkappa )=\frac{-2}{\varkappa ^{4} }\sqrt[3] {\left( \frac{2-\varkappa ^{3}}{\varkappa ^{3}}\right) ^{-2}}<0.$ So $g(\varkappa )$ is decreasing function on (0, 1]. Since $\{\underline{ \vartheta _{h_{\min }}}\}\le \{\underline{\vartheta _{h_{t}}}\}\le \{\underline{ \vartheta _{h_{\max }}}\}$ for all *t*. Then $g\left( \underline{\vartheta _{h_{\max }}}\right) \le g\left( \underline{\vartheta _{h_{t}}}\right) \le g\left( \underline{\vartheta _{h_{\min }}}\right)$ $(t=1,2,3,\ldots ,n)$ i.e.,
  $$\begin{aligned} \sqrt[3] {\frac{2-\left( \underline{\vartheta _{h_{\max }}}\right) ^{3}}{\left( \underline{\vartheta _{h_{\max }}}\right) ^{3}}}\le \sqrt[3] {\frac{2-\left( \underline{\vartheta _{h_{t}}}\right) ^{3}}{\left( \underline{\vartheta _{h_{t}}} \right) ^{3}}}\le \sqrt[3] {\frac{2-\left( \underline{\vartheta _{h_{\min }}} \right) ^{3}}{\left( \underline{\vartheta _{h_{\min }}}\right) ^{3}}} \end{aligned}$$
  and let $\varpi =\left( \varpi _{1},\varpi _{2},\ldots ,\varpi _{n}\right) ^{T}$ is weight vectors such that $\varpi _{t}\in [0,1]$ and $\oplus _{t=1}^{n}\varpi _{t}=1.$ We have
  3.1 $$\begin{aligned}{}&\Leftrightarrow \sqrt[3] {\overset{n}{\underset{t=1}{\otimes }}\left( \frac{ 2-\left( \underline{\vartheta _{h_{\max }}}\right) ^{3}}{\left( \underline{ \vartheta _{h_{\max }}}^{3}\right) }\right) ^{\varpi _{t}}}\le \sqrt[3] { \overset{n}{\underset{t=1}{\otimes }}\left( \frac{2-\left( \underline{\vartheta _{h_{t}}}\right) ^{3}}{\left( \underline{\vartheta _{h_{t}}}^{3}\right) } \right) ^{\varpi _{t}}} \nonumber \\&\le \sqrt[3] {\overset{n}{\underset{t=1}{\otimes }}\left( \frac{2-\left( \underline{\vartheta _{h_{\max }}}\right) ^{3}}{\left( \underline{\vartheta _{h_{\max }}}\right) ^{3}}\right) ^{\varpi _{t}}}\nonumber \\&\Leftrightarrow \sqrt[3] {\left( \frac{2-\left( \underline{\vartheta _{h_{\max }}}\right) ^{3}}{\left( \underline{\vartheta _{h_{\max }}}\right) ^{3}}\right) ^{\oplus _{t=1}^{n}\varpi _{t}}}\le \sqrt[3] {\overset{n}{\underset{t=1}{ \otimes }}\left( \frac{2-\left( \underline{\vartheta _{h_{t}}}\right) ^{3}}{ \left( \underline{\vartheta _{h_{t}}}\right) ^{3}}\right) ^{\varpi _{t}}} \nonumber \\&\le \sqrt[3] {\overset{n}{\underset{t=1}{\otimes }}\left( \frac{2-\left( \underline{\vartheta _{h_{\max }}}\right) ^{3}}{\left( \underline{\vartheta _{h_{\max }}}\right) ^{3}}\right) ^{\oplus _{t=1}^{n}\varpi _{t}}} \nonumber \\&\Leftrightarrow \underline{\vartheta _{h_{\min }}}\le \sqrt[3] {\overset{n}{ \underset{t=1}{\otimes }}\left( \frac{2-\left( \underline{\vartheta _{h_{t}}} \right) ^{3}}{\left( \underline{\vartheta _{h_{t}}}\right) ^{3}}\right) ^{\varpi _{t}}}\le \vartheta _{h_{\max }} \nonumber \\&\Leftrightarrow \underline{\vartheta _{h_{\min }}}\le \sqrt[3] {\overset{n}{ \underset{t=1}{\otimes }}\left( \frac{2-\left( \underline{\vartheta _{h_{t}}} \right) ^{3}}{\left( \underline{\vartheta _{h_{t}}}\right) ^{3}}\right) ^{\varpi _{t}}}\le \underline{\vartheta _{h_{\max }}} \end{aligned}$$
  Similarly, we can show that
  3.2 $$\begin{aligned} \Leftrightarrow \overline{\vartheta _{h_{\min }}}\le \sqrt[3] {\overset{n}{ \underset{t=1}{\otimes }}\left( \frac{2-\left( \overline{\vartheta _{h_{t}}} \right) ^{3}}{\left( \overline{\vartheta _{h_{t}}}\right) ^{3}}\right) ^{\varpi _{t}}}\le \overline{\vartheta _{h_{\max }}} \end{aligned}$$
  Again, let $f(y)=\sqrt[3] {\frac{1-y^{3}}{1+y^{3}}},$ $y\in [0,1].$ Then $f^{^{\prime }}(y)=\frac{-2y}{\left( 1+y^{3}\right) ^{3}}\sqrt[3] { \left( \frac{1-y^{3}}{1+y^{3}}\right) ^{-2}}<0.$ Thus *f*(*y*) is a decreasing function over [0, 1]. Since $\{\underline{\delta _{h_{\max }}}\}\le \{ \underline{\delta _{h_{t}}}\}\le \{\underline{\delta _{h_{\min }}}\}$ for all *t*. Then $g\left( \underline{\delta _{h_{\min }}}\right) \le g\left( \underline{\delta _{h_{t}}}\right) \le g\left( \underline{\delta _{h_{\max }}} \right)$ $(t=1,2,3,\ldots ,n)$ i.e.,
  $$\begin{aligned}{}&\Leftrightarrow \sqrt[3] {{\frac{1-\left( \underline{\delta _{h_{\min }}} \right) ^{3}}{1+\left( \underline{\delta _{h_{\min }}}\right) ^{3}}}}\le \sqrt[3] {{\frac{1-\left( \underline{\delta _{h_{t}}}\right) ^{3}}{1+\left( \underline{ \delta _{h_{t}}}\right) ^{3}}}} \\&\le \sqrt[3] {{\frac{1-\left( \underline{\delta _{h_{\max }}}\right) ^{3}}{ 1+\left( \underline{\delta _{h_{\max }}}\right) ^{3}}}}, (t=1,2,3,\ldots ,n) \end{aligned}$$
  and let $\varpi =\left( \varpi _{1},\varpi _{2},\ldots ,\varpi _{n}\right) ^{T}$ is weight vector such that $\varpi _{t}\in [0,1]$ and $\oplus _{t=1}^{n}\varpi _{t}=1,$ we have
  3.3 $$\begin{aligned}{}&\Leftrightarrow \sqrt[3] {{\overset{n}{\underset{t=1}{\otimes }}\left( \frac{ 1-\left( \underline{\delta _{h_{\min }}}\right) ^{3}}{1+\left( \underline{\delta _{h_{\min }}}\right) ^{3}}\right) ^{\varpi _{t}}}}\le \sqrt[3] { {\overset{n}{ \underset{t=1}{\otimes }}\left( \frac{1-\left( \underline{\delta _{h_{t}}} \right) ^{3}}{1+\left( \underline{\delta _{h_{t}}}\right) ^{3}}\right) ^{\varpi _{t}}}} \nonumber \\&\le \sqrt[3] { {\overset{n}{\underset{t=1}{\otimes }}\left( \frac{1-\left( \underline{\delta _{h_{\max }}}\right) ^{3}}{1+\left( \underline{\delta _{h_{\max }}}\right) ^{3}}\right) ^{\varpi _{t}}}}, \nonumber \\&\Leftrightarrow \sqrt[3] { {\left( \frac{1-\left( \underline{\delta _{h_{\min }}} \right) ^{3}}{1+\left( \underline{\delta _{h_{\min }}}\right) ^{3}}\right) ^{\oplus _{t=1}^{n}\varpi _{t}}}}\le \sqrt[3] {{\overset{n}{\underset{t=1}{ \otimes }}\left( \frac{1-\left( \underline{\delta _{h_{t}}}\right) ^{3}}{ 1+\left( \underline{\delta _{h_{t}}}\right) ^{3}}\right) ^{\varpi _{t}}}} \nonumber \\&\le \sqrt[3] { {\left( \frac{1-\left( \underline{\delta _{h_{\max }}}\right) ^{3}}{1+\left( \underline{\delta _{h_{\max }}}\right) ^{3}}\right) ^{\oplus _{t=1}^{n}\varpi _{t}}}}, \end{aligned}$$
  3.4 $$\begin{aligned}{}&\Leftrightarrow \delta _{h_{\max }}\le \sqrt[3]{ {\overset{n}{\underset{t=1}{ \otimes }}\left( \frac{1-\left( \underline{\delta _{h_{t}}}\right) ^{3}}{ 1+\left( \underline{\delta _{h_{t}}}\right) ^{3}}\right) ^{\varpi _{t}}}}\le \underline{\delta _{h_{\min }}}, \nonumber \\&\Leftrightarrow \underline{\delta _{h_{\max }}}\le \sqrt[3] {{\overset{n}{ \underset{t=1}{\otimes }}\left( \frac{1-\left( \underline{\delta _{h_{t}}} \right) ^{3}}{1+\left( \underline{\delta _{h_{t}}}\right) ^{3}}\right) ^{\varpi _{t}}}}\le \underline{\delta _{h_{\min }}}, \end{aligned}$$
  In a similar way, we can show that
  3.5 $$\begin{aligned} \Leftrightarrow \overline{\delta _{h_{\max }}}\le \sqrt[3] {\overset{n}{ \underset{t=1}{\otimes }}\left( \frac{2-\left( \overline{\delta _{h_{t}}} \right) ^{3}}{\left( \overline{\delta _{h_{t}}}\right) ^{3}}\right) ^{\varpi _{t}}}\le \overline{\delta _{h_{\min }}} \end{aligned}$$
  By routine calculations, we can show the aforementioned results for $q>3.$ Thus from (3.1), (3.2), (3.4) and (3.5) we have
  $$\begin{aligned} \left( \mathcal {Y} (\varrho )\right) ^{-}\le q-ROHFREWG\left( \mathcal {Y} (\varrho _{1}),\mathcal {Y} (\varrho _{2}),\ldots ,\mathcal {Y} (\varrho _{n})\right) \le \left( \mathcal {Y} (\varrho )\right) ^{+}. \end{aligned}$$
(3): **Monotonicity:** The proof is similar to the proof of (2).

$$\square$$

### The *q*-Rung orthopair hesitant fuzzy rough Einstein ordered weighted geometric aggregation operator

In this subsection, the *q*-ROHFREOWG aggregation operator is introduced, and the key characteristics of the proposed operator are demonstrated.

#### Definition 3.4

Let $\mathcal {Y} (\varrho _{t})=(\underline{\mathcal {Y} }(\varrho _{t}),\overline{\mathcal {Y} }(\varrho _{t}))$ $(t=1,2,3,4,\ldots ,n)$ be the collection of *q*-ROHFR values then *q*-ROHFREOWG operator is determined as:

$$\begin{aligned} q-ROHFREOWG\left( \mathcal {Y} (\varrho _{1}),\mathcal {Y} (\varrho _{2}),\ldots ,\mathcal {Y} (\varrho _{n})\right) =\left( \bigoplus \limits _{t=1}^{n}\left( \underline{\mathcal {Y} _{\rho }}(\varrho _{t})\right) ^{\varpi _{t}},\bigoplus \limits _{t=1}^{n}\left( \overline{\mathcal {Y} _{\rho }}(\varrho _{t})\right) ^{\varpi _{t}}\right) , \end{aligned}$$

where $\varpi =\left( \varpi _{1},\varpi _{2},\ldots ,\varpi _{n}\right) ^{T}$ is the weights vector such that $\bigoplus \nolimits _{t=1}^{n}\varpi _{t}=1$ and $0\le$ $\varpi _{t}\le 1.$

#### Theorem 3.5

*Let* $\mathcal {Y} (\varrho _{t})=(\underline{\mathcal {Y} }(\varrho _{t}),\overline{\mathcal {Y} }(\varrho _{t}))$ $(t=1,2,3,\ldots ,n)$ *be the collection of* *q-ROHFR values with weights vector* $\varpi =\left( \varpi _{1},\varpi _{2},\ldots ,\varpi _{n}\right) ^{T}$ *such that* $\bigoplus \nolimits _{t=1}^{n}\varpi _{t}=1$ *and* $0\le$ $\varpi _{t}\le 1.$ *Then* *q-ROHFREOWG operator is described as:*

$$\begin{aligned}&q-ROHFREOWG\left( \mathcal {Y} (\varrho _{1}),\mathcal {Y} (\varrho _{2}),\ldots ,\mathcal {Y} (\varrho _{n})\right) \\&\quad =\left( \overset{n}{\underset{t=1}{\otimes }}\left( \underline{ \mathcal {Y} _{\rho }}(\varrho _{t})\right) ^{\varpi _{t}},~\overset{n}{\underset{t=1}{ \otimes }}\left( \overline{\mathcal {Y}_{\rho }}(\varrho _{t})\right) ^{\varpi _{t}}\right) \\&\quad =\left( \begin{array}{c} \text { }\left\{ \bigcup \limits _{\underline{\vartheta _{h_{t}}}\in \eth _{h_{ \underline{\mathcal {Y} _{\rho }}(\varrho )}}}\left( \frac{\sqrt[q] {2\overset{n}{ \underset{t=1}{\otimes }}\left( \underline{\vartheta _{\rho _{h_{t}}}} ^{q}\right) ^{\varpi _{t}}}}{\sqrt[q] {\overset{n}{\underset{t=1}{\otimes }} \left( 2-\underline{\vartheta {\rho {h_{t}}}}^{q}\right) ^{\varpi _{t}}+ \overset{n}{\underset{t=1}{\otimes }}\left( \underline{\vartheta {\rho _{h_{t}}}}^{q}\right) ^{\varpi _{t}}}}\right) ,\bigcup \limits _{\underline{ \delta {h_{t}}}\in \psi _{h_{\underline{\mathcal {Y}{\rho }}(\varrho )}}}\left( \frac{ \sqrt[q] {\overset{n}{\underset{t=1}{\otimes }}\left( 1+\underline{\delta {\rho _{h_{t}}}^{q}}\right) ^{\varpi _{t}}-\overset{n}{\underset{t=1}{\otimes }} \left( 1-\underline{\delta {\rho _{h_{t}}}^{q}}\right) ^{\varpi _{t}}}}{\sqrt[q] {\overset{n}{\underset{t=1}{\otimes }}\left( 1+\underline{\delta _{\rho _{h_{t}}}^{q}}\right) ^{\varpi _{t}}+\overset{n}{\underset{t=1}{\otimes }} \left( 1-\underline{\delta {\rho {h_{t}}}^{q}}\right) ^{\varpi _{t}}}}\right) \right\} \\ \left\{ \bigcup \limits _{\overline{\vartheta _{h_{t}}}\in \psi _{h_{\overline{ \mathcal {Y} _{\rho }}(\varrho )}}}\left( \frac{\sqrt[q] {2\overset{n}{\underset{t=1}{ \otimes }}\left( \overline{\vartheta _{\rho _{h_{t}}}^{q}}\right) ^{\varpi _{t}} }}{\sqrt[q] {\overset{n}{\underset{t=1}{\otimes }}\left( 2-\overline{\vartheta _{\rho _{h_{t}}}^{q}}\right) ^{\varpi _{t}}+\overset{n}{\underset{t=1}{ \otimes }}\left( \overline{\vartheta _{\rho _{h_{t}}}^{q}}\right) ^{\varpi _{t}} }}\right) ,\bigcup \limits _{\overline{\delta _{h_{t}}}\in \psi _{h_{\overline{ \mathcal {Y} }_{\rho }(\varrho )}}}\left( \frac{\sqrt[q] {\overset{n}{\underset{t=1}{ \otimes }}\left( 1+\overline{\delta _{\rho _{h_{t}}}^{q}}\right) ^{\varpi _{t}}- \overset{n}{\underset{t=1}{\otimes }}\left( 1-\overline{\delta _{\rho _{h_{t}}}^{q}}\right) ^{\varpi _{t}}}}{\sqrt[q] {\overset{n}{\underset{t=1}{ \otimes }}\left( 1+\overline{\delta _{\rho _{h_{t}}}^{q}}\right) ^{\varpi _{t}}+ \overset{n}{\underset{t=1}{\otimes }}\left( 1-\overline{\delta _{\rho _{h_{t}}}^{q}}\right) ^{\varpi _{t}}}}\right) \right\} \end{array} \right) , \end{aligned}$$

*where* $\varpi =\left( \varpi _{1},\varpi _{2},\ldots ,\varpi _{n}\right) ^{T}$ *is the weight vector such that* $\oplus _{t=1}^{n}\varpi _{t}=1$ *and* $0\le$ $\varpi _{t}\le 1.$

#### Proof

The proof is straightforward and is similar to Theorem 3.2. $\square$

#### Theorem 3.6

*Let* $\mathcal {Y} (\varrho _{t})=(\underline{\mathcal {Y} }(\varrho _{t}),\overline{\mathcal {Y} }(\varrho _{t}))$ $(t=1,2,3,\ldots ,n)$ *be the collection of* *q-ROHFR values and* $\varpi =\left( \varpi _{1},\varpi _{2},\ldots ,\varpi _{n}\right) ^{T}$ *is the weight vector such that* $\varpi _{t}\in [0,1]$ *and* $\oplus _{t=1}^{n}\varpi _{t}=1.$ *Then* *q-ROHFREOWG operator satisfy the following properties:*

(1): **Idempotency: ***If* $\mathcal {Y} (\varrho _{t})=\mathfrak {F} (\varrho )$ *for* $t=1,2,3,\ldots ,n$ *where* $\mathfrak {F} (\varrho )=\left( \underline{\mathfrak {F} } (\varrho ),\overline{\mathfrak {F} }(\varrho )\right) =\left( (\underline{b_{h(x)}}, \underline{d_{h(x)}}),(\overline{b}_{h(x)},\overline{d_{h(x)}})\right) .$ *Then*
  $$\begin{aligned} q-ROHFREOWG\left( \mathcal {Y} (\varrho _{1}),\mathcal {Y} (\varrho _{2}),\ldots ,\mathcal {Y} (\varrho _{n})\right) =\mathfrak {F} (\varrho ). \end{aligned}$$
(2): **Boundedness: ***Let* $\left( \mathcal {Y} (\varrho )\right) _{\min }=\left( \underset{t}{\min }\underline{\mathcal {Y} }\left( \varrho _{t}\right) ,\underset{t}{ \max }\overline{\mathcal {Y} }(\varrho _{t})\right)$ and $\left( \mathcal {Y} (\varrho )\right) _{\max }=$ $\left( \underset{t}{\max }\underline{\mathcal {Y} }\left( \varrho _{t}\right) ,\underset{t}{\min }\overline{\mathcal {Y} }(\varrho _{t})\right) .$ *Then*
  $$\begin{aligned} \left( \mathcal {Y} (\varrho )\right) _{\min }\le q-ROHFREOWG\left( \mathcal {Y} (\varrho _{1}),\mathcal {Y} (\varrho _{2}),\ldots ,\mathcal {Y} (\varrho _{n})\right) \le \left( \mathcal {Y} (\varrho )\right) _{\max }. \end{aligned}$$
(3): **Monotonicity: ***Suppose* $\mathfrak {F} (\varrho )=\left( \underline{\mathfrak {F} }(\varrho _{t}),\overline{\mathfrak {F} }(\varrho _{t})\right) (t=1,2,\ldots ,n)$*is another collection of* *q-ROHFR values such that* $\underline{\mathfrak {F} }(\varrho _{t})\le \underline{\mathcal {Y} }\left( \varrho _{t}\right)$ *and* $\overline{\mathfrak {F} }(\varrho _{t})\le \overline{\mathcal {Y} } (\varrho _{t})$. *Then*
  $$\begin{aligned} q-ROHFREOWG\left( \mathfrak {F} (\varrho _{1}),\mathfrak {F} (\varrho _{2}),\ldots ,\mathfrak {F} (\varrho _{n})\right) \le q-ROHFREOWG\left( \mathcal {Y} (\varrho _{1}),\mathcal {Y} (\varrho _{2}),\ldots \mathcal {Y} (\varrho _{n})\right) . \end{aligned}$$

#### Proof

The proof is straightforward and is similar to Theorem 3.3. $\square$

### The *q*-rung orthopair hesitant fuzzy rough Einstein hybrid geometric aggregation operator

In this part a  *q*-ROHFRHWG aggregation operator is introduce, as well as the essential properties of the suggested operators are addressed.

#### Definition 3.7

Let $\mathcal {Y} (\varrho _{t})=(\underline{\mathcal {Y} }(\varrho _{t}),\overline{\mathcal {Y} }(\varrho _{t}))$ $(t=1,2,3,4,\ldots ,n)$ be the collection of *q*-ROHFR values and let $\varpi =\left( \varpi _{1},\varpi _{2},\ldots ,\varpi _{n}\right) ^{T}$ is the weights vector of the given collection of *q*-ROHFR values such that $\bigoplus \nolimits _{t=1}^{n}\varpi _{t}=1$ and $0\le$ $\varpi _{t}\le 1.~$Let $\left( w_{1},w_{2},\ldots ,w_{n}\right) ^{T}~$be the associated weights such that $\bigoplus \nolimits _{t=1}^{n}w_{t}=1$ and $0\le$ $w_{t}\le 1.$ Then the *q*-ROHFREHWG operator is determined as:

$$\begin{aligned} q-ROHFREHWG\left( \mathcal {Y} (\varrho _{1}),\mathcal {Y} (\varrho _{2}),\ldots ,\mathcal {Y} (\varrho _{n})\right) =\left( \bigoplus \limits _{t=1}^{n}\left( \widehat{\underline{ \mathcal {Y} _{\rho }}}(\varrho _{t})\right) ^{\varpi _{t}},\bigoplus \limits _{t=1}^{n}\left( \overline{\widehat{\mathcal {Y} }_{\rho }} (\varrho _{t})\right) ^{\varpi _{t}}\right) , \end{aligned}$$

where $$\left( \widehat{\underline{\mathcal {Y} }_{\rho }}(\varrho _{t}),\overline{ \widehat{\mathcal {Y} }}\rho (\varrho _{t})\right) =\left( n\varpi _{t}\underline{\mathcal {Y} } _{\rho }(\varrho _{t}),n\varpi _{t}\overline{\mathcal {Y} }\rho (\varrho _{t})\right) .$$

#### Theorem 3.8

*Let* $\mathcal {Y} (\varrho _{t})=(\underline{\mathcal {Y} }(\varrho _{t}),\overline{\mathcal {Y} }(\varrho _{t}))$ $(t=1,2,3,4,\ldots ,n)$ *be the collection of* *q-ROHFR values and let* $\varpi =\left( \varpi _{1},\varpi _{2},\ldots ,\varpi _{n}\right) ^{T}~$ *be the weights vector such that* $\bigoplus \nolimits _{t=1}^{n}w_{t}=1$ *and* $0\le$ $w_{t}\le 1.$ *Let* $\left( w_{1},w_{2},\ldots ,w_{n}\right) ^{T}$ *is the associated weights of the given collection of* *q-ROHFR values such that* $\bigoplus \nolimits _{t=1}^{n}\varpi _{t}=1$ *and* $0\le$ $\varpi _{t}\le 1.~$ *Then the* *q-ROHFREHWG operator is described as:*

$$\begin{aligned}&q-ROHFREHWG\left( \mathcal {Y} (\varrho _{1}),\mathcal {Y} (\varrho _{2}),\ldots ,\mathcal {Y} (\varrho _{n})\right) \\&\quad =\left( \overset{n}{\underset{t=1}{\otimes }}\left( \underline{ \widehat{\mathcal {Y} _{\rho }}}(\varrho _{t})\right) ^{w_{t}},\overset{n}{\underset{t=1 }{\otimes }}\left( \overline{\widehat{\mathcal {Y} }_{\rho }}(\varrho _{t})\right) ^{w_{t}}\right) \\&\quad =\left( \begin{array}{c} \text { }\left\{ \bigcup \limits _{\underline{\vartheta _{h_{t}}}\in \eth _{h_{ \widehat{\underline{\mathcal {Y} _{\rho }}}(\varrho )}}}\left( \frac{\sqrt[q] {2\overset{ n}{\underset{t=1}{\otimes }}\left( \underline{\widehat{\vartheta }_{\rho _{h_{t}}}}^{q}\right) ^{w_{t}}}}{\sqrt[q] {\overset{n}{\underset{t=1}{\otimes }}\left( 2-\underline{\widehat{\vartheta }_{\rho _{h_{t}}}}^{q}\right) ^{w_{t}}+ \overset{n}{\underset{t=1}{\otimes }}\left( \underline{\widehat{\vartheta } _{\rho _{h_{t}}}}^{q}\right) ^{w_{t}}}}\right) ,\bigcup \limits _{\underline{ \delta _{h_{t}}}\in \psi _{h_{\underline{\widehat{\mathcal {Y} _{\rho }}}(\varrho )}}}\left( \frac{\sqrt[q] {\overset{n}{\underset{t=1}{\otimes }}\left( 1+ \underline{\widehat{\delta }_{\rho _{h_{t}}}^{q}}\right) ^{w_{t}}-\overset{n}{ \underset{t=1}{\otimes }}\left( 1-\underline{\widehat{\delta }_{\rho _{h_{t}}}^{q}}\right) ^{w_{t}}}}{\sqrt[q] {\overset{n}{\underset{t=1}{\otimes }}\left( 1+\underline{\widehat{\delta }_{\rho _{h_{t}}}^{q}}\right) ^{w_{t}}+ \overset{n}{\underset{t=1}{\otimes }}\left( 1-\underline{\widehat{\delta } _{\rho _{h_{t}}}^{q}}\right) ^{w_{t}}}}\right) \right\} \\ \left\{ \bigcup \limits _{\overline{\vartheta _{h_{t}}}\in \psi _{h_{\overline{ \widehat{\mathcal {Y} }_{\rho }}(\varrho )}}}\left( \frac{\sqrt[q] {2\overset{n}{ \underset{t=1}{\otimes }}\left( \overline{\widehat{\vartheta }_{\rho _{h_{t}}}^{q}}\right) ^{w_{t}}}}{\sqrt[q] {\overset{n}{\underset{t=1}{\otimes }}\left( 2-\overline{\widehat{\vartheta }_{\rho _{h_{t}}}^{q}}\right) ^{w_{t}}+ \overset{n}{\underset{t=1}{\otimes }}\left( \overline{\widehat{\vartheta } _{\rho _{h_{t}}}^{q}}\right) ^{w_{t}}}}\right) ,\bigcup \limits _{\overline{ \delta _{h_{t}}}\in \psi _{h_{\overline{\widehat{\mathcal {Y} }_{\rho }}(\varrho )}}}\left( \frac{\sqrt[q] {\overset{n}{\underset{t=1}{\otimes }}\left( 1+ \overline{\widehat{\delta }_{\rho _{h_{t}}}^{q}}\right) ^{w_{t}}-\overset{n}{ \underset{t=1}{\otimes }}\left( 1-\overline{\widehat{\delta }_{\rho _{h_{t}}}^{q}}\right) ^{w_{t}}}}{\sqrt[q] {\overset{n}{\underset{t=1}{\otimes }}\left( 1+\overline{\widehat{\delta }_{\rho _{h_{t}}}^{q}}\right) ^{w_{t}}+ \overset{n}{\underset{t=1}{\otimes }}\left( 1-\overline{\widehat{\delta }_{\rho _{h_{t}}}^{q}}\right) ^{w_{t}}}}\right) \right\} \end{array} \right) , \end{aligned}$$

#### Proof

The proof is straightforward and is similar to Theorem 3.2. $\square$

#### Theorem 3.9

*Let* $\mathcal {Y} (\varrho _{t})=(\underline{\mathcal {Y} }(\varrho _{t}),\overline{\mathcal {Y} }(\varrho _{t}))$ $(t=1,2,3,4,\ldots ,n)$ *be the collection of* *q-ROHFR values and let* $\left( w_{1},w_{2},\ldots ,w_{n}\right) ^{T}$ *be the associated weights such that* $\bigoplus \nolimits _{t=1}^{n}w_{t}=1$ *and* $0\le$ $w_{t}\le 1.$ *Let* $\varpi =\left( \varpi _{1},\varpi _{2},\ldots ,\varpi _{n}\right) ^{T}$ *be the weights vector of the given collection of* *q-ROHFR values such that* $\bigoplus \nolimits _{t=1}^{n}\varpi _{t}=1$ *and* $0\le$ $\varpi _{t}\le 1.$ *Then* *q-ROHFREHWG operator satisfy the following properties:*

(1): **Idempotency: ***If* $\mathcal {Y} (\varrho _{t})=\mathfrak {F} (\varrho )$ *for* $t=1,2,3,\ldots ,n$ *where* $\mathfrak {F} (\varrho )=\left( \underline{\mathfrak {F} } (\varrho ),\overline{\mathfrak {F} }(\varrho )\right) =\left( (\underline{b_{h(x)}}, \underline{d_{h(x)}}),(\overline{b}_{h(x)},\overline{d_{h(x)}})\right) .$ *Then*
  $$\begin{aligned} q-ROHFREHWG\left( \mathcal {Y} (\varrho _{1}),\mathcal {Y} (\varrho _{2}),\ldots ,\mathcal {Y} (\varrho _{n})\right) =\mathfrak {F} (\varrho ). \end{aligned}$$
(2): **Boundedness: ***Let* $\left( \mathcal {Y} (\varrho )\right) _{\min }=\left( \underset{t}{\min }\underline{\mathcal {Y} }\left( \varrho _{t}\right) ,\underset{t}{ \max }\overline{\mathcal {Y} }(\varrho _{t})\right)$ and $\left( \mathcal {Y} (\varrho )\right) _{\max }=$ $\left( \underset{t}{\max }\underline{\mathcal {Y} }\left( \varrho _{t}\right) ,\underset{t}{\min }\overline{\mathcal {Y} }(\varrho _{t})\right) .$ *Then*
  $$\begin{aligned} \left( \mathcal {Y} (\varrho )\right) _{\min }\le q-ROHFREHWG\left( \mathcal {Y} (\varrho _{1}),\mathcal {Y} (\varrho _{2}),\ldots ,\mathcal {Y} (\varrho _{n})\right) \le \left( \mathcal {Y} (\varrho )\right) _{\max }. \end{aligned}$$
(3): **Monotonicity: ***Suppose* $\mathfrak {F} (\varrho )=\left( \underline{\mathfrak {F} }(\varrho _{t}),\overline{\mathfrak {F} }(\varrho _{t})\right) (t=1,2,\ldots ,n)$ *is another collection of* *q-ROHFR values such that* $\underline{\mathfrak {F} }(\varrho _{t})\le \underline{\mathcal {Y} }\left( \varrho _{t}\right)$ *and* $\overline{\mathfrak {F} }(\varrho _{t})\le \overline{\mathcal {Y} } (\varrho _{t})$. *Then*
  $$\begin{aligned} q-ROHFREHWG\left( \mathfrak {F} (\varrho _{1}),\mathfrak {F} (\varrho _{2}),\ldots ,\mathfrak {F} (\varrho _{n})\right) \le q-ROHFREHWG\left( \mathcal {Y} (\varrho _{1}),\mathcal {Y} (\varrho _{2}),\ldots ,\mathcal {Y} (\varrho _{n})\right) . \end{aligned}$$

#### Proof

The proof is easy and is similar to the proof of Theorem 3.3. $\square$

## The multi-attribute decision making methodology

In this section, we developed an approach to dealing with uncertainty in MAGDM using *q*-ROHFR information. Consider a DM problem with a set $\left\{ A_{1},A_{2},\ldots ,A_{n}\right\}$ of *n* alternatives and a set of *n* attributes $\left\{ \chi _{1},\chi _{2},\ldots ,\chi _{n}\right\}$ with $(\varpi _{1},\varpi _{2},\ldots ,\varpi _{n})^{T}$ the weights, that is, $\varpi _{t}\in [0,1]$, $\oplus _{t=1}^{n}\varpi _{t}=1.$ To test the reliability of kth alternative $A_{t}$ under the the attribute $\chi _{t},$ let $\left\{ \mathring{D}_{1},\mathring{D}_{2},\ldots ,\mathring{D}_{\hat{\jmath } }\right\}$ be a set of decision makers (DMs). The expert evaluation matrix is defined as follows:

$$\begin{aligned} M= & {} \left[ \overline{\mathcal {Y} }(\varrho _{tj}^{\hat{\jmath }})\right] _{m\times n}\\= & {} \left[ \begin{array}{cccc} \left( \underline{\mathcal {Y} }(\varrho _{11}),\overline{\mathcal {Y} }(\varrho _{11})\right) &{} \left( \underline{\mathcal {Y} }(\varrho _{12}),\overline{\mathcal {Y} }(\varrho _{12})\right) &{} \cdots &{} \left( \underline{\mathcal {Y} }(\varrho _{1j}),\overline{\mathcal {Y} }(\varrho _{1j})\right) \\ \left( \underline{\mathcal {Y} }(\varrho _{21}),\overline{\mathcal {Y} }(\varrho _{21})\right) &{} \left( \underline{\mathcal {Y} }(\varrho _{22}),\overline{\mathcal {Y} }(\varrho _{22})\right) &{} \cdots &{} \left( \underline{\mathcal {Y} }(\varrho _{2j}),\overline{\mathcal {Y} }(\varrho _{2j})\right) \\ \left( \underline{\mathcal {Y} }(\varrho _{31}),\overline{\mathcal {Y} }(\varrho _{31})\right) &{} \left( \underline{\mathcal {Y} }(\varrho _{32}),\overline{\mathcal {Y} }(\varrho _{32})\right) &{} \cdots &{} \left( \underline{\mathcal {Y} }(\varrho _{3j}),\overline{\mathcal {Y} }(\varrho _{3j})\right) \\ \vdots &{} \vdots &{} \ddots &{} \vdots \\ \left( \underline{\mathcal {Y} }(\varrho _{t1}),\overline{\mathcal {Y} }(\varrho _{t1})\right) &{} \left( \underline{\mathcal {Y} }(\varrho _{t2}),\overline{\mathcal {Y} }(\varrho _{t2})\right) &{} \cdots &{} \left( \underline{\mathcal {Y} }(\varrho _{tj}),\overline{\mathcal {Y} }(\varrho _{tj})\right) \end{array} \right] , \end{aligned}$$

where

$$\begin{aligned} \underline{\mathcal {Y} }(\varrho )=\left\{ \left\langle \mu ,\eth _{h_{ \underline{\mathcal {Y} }(\varrho )}}(\mu ),\psi _{h_{\underline{\mathcal {Y} } (\varrho )}}(\mu )\right\rangle |\mu \in \Im \right\} \end{aligned}$$

and

$$\begin{aligned} \overline{\mathcal {Y} }(\varrho _{tj})= & {} \left\{ \left\langle \mu ,\eth _{h_{ \overline{\mathcal {Y} }(\varrho )}}(\mu ),\psi _{h_{\overline{\mathcal {Y} }(\varrho )}}( \mu )\right\rangle |\mu \in \Im \right\} \\ 0\le & {} \left( \max (\eth _{h_{\overline{\mathcal {Y} }(\varrho )}}(\mu ))\right) ^{q}+\left( \min (\psi _{h_{\overline{\mathcal {Y} }(\varrho )}}(\mu ))\right) ^{q}\le 1 \end{aligned}$$

and

$$\begin{aligned} 0\le \left( \min (\eth _{h_{\underline{\mathcal {Y} }(\varrho )}}(\mu )\right) ^{q}+\left( \max (\psi _{h_{\underline{\mathcal {Y}}(\varrho )}}(\mu ))\right) ^{q}\le 1, \end{aligned}$$

are the *q*-ROHFR values. The following are the main steps for MAGDM:

**Step-1**: Construct the experts evaluation matrices as
  $$\begin{aligned} \left( E\right) ^{_{\hat{\jmath }}}=\left[ \begin{array}{cccc} \left( \underline{\mathcal {Y} }(\varrho _{11}^{_{\hat{\jmath }}}),\overline{\mathcal {Y} } (\varrho _{11}^{_{\hat{\jmath }}})\right) &{} \left( \underline{\mathcal {Y} }(\varrho _{12}^{_{\hat{\jmath }}}),\overline{\mathcal {Y} }(\varrho _{12}^{_{\hat{\jmath } }})\right) &{} \cdots &{} \left( \underline{\mathcal {Y} }(\varrho _{1j}^{_{\hat{ \jmath }}}),\overline{\mathcal {Y} }(\varrho _{1j}^{_{\hat{\jmath }}})\right) \\ \left( \underline{\mathcal {Y} }(\varrho _{21}^{_{\hat{\jmath }}}),\overline{\mathcal {Y} } (\varrho _{21}^{_{\hat{\jmath }}})\right) &{} \left( \underline{\mathcal {Y} }(\varrho _{22}^{_{\hat{\jmath }}}),\overline{\mathcal {Y} }(\varrho _{22}^{_{\hat{\jmath } }})\right) &{} \cdots &{} \left( \underline{\mathcal {Y} }(\varrho _{2j}^{_{\hat{ \jmath }}}),\overline{\mathcal {Y} }(\varrho _{2j}^{_{\hat{\jmath }}})\right) \\ \left( \underline{\mathcal {Y} }(\varrho _{31}^{_{\hat{\jmath }}}),\overline{\mathcal {Y} } (\varrho _{31}^{_{\hat{\jmath }}})\right) &{} \left( \underline{\mathcal {Y} }(\varrho _{32}^{_{\hat{\jmath }}}),\overline{\mathcal {Y} }(\varrho _{32}^{_{\hat{\jmath } }})\right) &{} \cdots &{} \left( \underline{\mathcal {Y} }(\varrho _{3j}^{_{\hat{ \jmath }}}),\overline{\mathcal {Y} }(\varrho _{3j}^{_{\hat{\jmath }}})\right) \\ \vdots &{} \vdots &{} \ddots &{} \vdots \\ \left( \underline{\mathcal {Y} }(\varrho _{t1}^{_{\hat{\jmath }}}),\overline{\mathcal {Y} } (\varrho _{t1}^{_{\hat{\jmath }}})\right) &{} \left( \underline{\mathcal {Y} }(\varrho _{t2}^{_{\hat{\jmath }}}),\overline{\mathcal {Y} }(\varrho _{t2}^{_{\hat{\jmath } }})\right) &{} \cdots &{} \left( \underline{\mathcal {Y} }(\varrho _{tj}^{_{\hat{ \jmath }}}),\overline{\mathcal {Y} }(\varrho _{tj}^{_{\hat{\jmath }}})\right) \end{array} \right] \end{aligned}$$
  where $\hat{\jmath }$ shows the number of experts.
**Step-2**: Explore the expert matrices that were normalised $\left( N\right) ^{\hat{\jmath }},$ as
  $$\begin{aligned} \left( N\right) ^{\hat{\jmath }}= \begin{array}{ccc} \mathcal {Y} (\varrho _{tj})=\left( \underline{\mathcal {Y} }\left( \varrho _{tj}\right) , \overline{\mathcal {Y} }\left( \varrho _{tj}\right) \right) &{} \text {if} &{} \, \text { for benefit} \\ \left( \mathcal {Y} (\varrho _{tj})\right) ^{c}=\left( \left( \underline{\mathcal {Y} }\left( \varrho _{tj}\right) \right) ^{c},\left( \overline{\mathcal {Y} }\left( \varrho _{tj}\right) \right) ^{c}\right) &{} \text {if} &{} \text {for cost } \end{array} \end{aligned}$$
**Step-3**: Using the suggested aggregation information, compute the *q*-ROHFR values for each considered alternative with respect to the given list of criteria/attributes.
**Step-4**: Determine the ranking of alternatives based on the score function as follows:
  $$\begin{aligned} SR(\mathcal {Y} (\varrho ))=\frac{1}{4}\left( \begin{array}{c} 2+\frac{1}{M_{\mathcal {F}}}\sum \limits _{\underline{\vartheta _{h_{t}}}\in \eth _{h_{\underline{\bowtie }(\varrho )}}}(\underline{\vartheta _{h_{t}}})+ \frac{1}{N_{\mathcal {F}}}\sum \limits _{\overline{\vartheta _{h_{t}}}\in \psi _{h_{\overline{\mathcal {Y} }(\varrho )}}}(\overline{\vartheta _{h_{t}}}) \\ \frac{1}{M_{\mathcal {F}}}\sum \limits _{\underline{\delta _{h_{t}}}\in \psi _{h_{ \underline{\bowtie }(\varrho )}}}(\underline{\delta _{h_{t}}})-\frac{1}{M_{ \mathcal {F}}}\sum \limits _{\overline{\delta _{h_{t}}}\in \psi _{h_{\overline{ \mathcal {Y} }(\varrho )}}}(\overline{\delta _{h_{t}}}) \end{array} \right) . \end{aligned}$$
**Step-5**: All alternative scores must be ranked in descending order. The superior/best alternative will be the one with a higher value.

## The application of proposed decision-making approach

To demonstrate the validity of the established operators, we present a numerical MCGDM example that use the suggested aggregations technique in combination with *q*-ROHFR information to identify the optimum location for a wind power plant.

### Case study (the evaluation of wind power station site selection)

Currently, the civilization is facing threat because of several environmental issues caused by the fossil fuel consumption. As a result, several renewable energy power generation projects have gained a prominent development. Renewable energy is the most cost-effective and environmentally safe energy source which is never going to exhaus^88^. Renewable energy generation is a burgeoning field, with more and more renewable energy sources being investigated and it has a bright future. Therefore, nations have made significant investments in renewable energy power generation^90^. More comprehensive review techniques are needed to choose the appropriate initiatives, so that we can identify their strengths and weaknesses and put forward some new suggestions to accomplish the objectives. The site selection is usually a crucial challenge in dealing with all renewable energy projects for professional and decision-makers because several factors were evaluated while deciding on a location for a large-scale renewable energy installation^92^. The aim is to optimize the location in which the power will produce in more efficient and cost-effective systems and fulfil the demand while maintaining a minimal impact on the environment and society.Wind energy stations are among the most efficient and environmentally energy sources, making a significant contribution to existing energy supply. It is essential to mention that the installation of a wind power plant requires wind energy potential, distance from the electricity grid, distance to roads and urban areas.

(1): **Wind energy potential**: The most significant aspect is the wind energy potential criteria. The average wind speed in the region is a fundamental requirement for the economic efficiency of wind generators. Winds are highly influenced and reformed by plant, water bodies, spatial patterns, local topography, weather changes, and a variety of other factors.
(2): **Slope of topography**: The slope of a site is a significant economic and transportation factor. Wind energy power plant should ideally be erected on completely flat land. However, if this is not accessible, the slope must be developed, which will take effort and time, raising installation costs.
(3): **Range from the energy grid:** It is intrinsically connected to the efficiency of energy transmission to power grids or transformers, because the shorter the range and the less energy spent, the closer to the energy stations. Additionally, shorter ranges lead to lower network connection costs.
(4): **Distance from roads and urban areas**: Wind farms particularly in the urban and high-consumption regions provide financial benefits. When wind turbines are located near areas with significant energy use, the energy produced by the plantation will require minimum transmission lines to transfer the power, minimising the cost of transmitting the energy to consumers.

### The evaluation procedure of a site selection for wind power station

Assume an organization intends to evaluate the selection process of a location for a wind power project. They will appoint a team of specialists to choose the best location for a wind power plant. In this problem, we analyse a case study for selecting site in which four alternative locations, say, $\left\{ A_{1},A_{2},A_{3},A_{4}\right\}$ are evaluated in addressing the problem and we must select the ideal one. Let $\left\{ \chi _{1},\chi _{2},\chi _{3},\chi _{4}\right\}$ be the attributes of each alternative based on the influencing factors determined as follows: wind energy potential $\left( \chi _{1}\right)$, slope of topography $\left( \chi _{2}\right)$, distance from the electricity grid $\left( \chi _{3}\right)$ and distance from roads and urban areas $\left( \chi _{4}\right)$ of wind power site. Because of the uncertainty, the DMs’ selection information is presented as *q*-ROHFR information. The weights vector for criteria is $\varpi =\left( 0.13,0.27,0.29,0.31\right) ^{T}$. To solve the DM problem using the developed methodology for evaluating alternatives, the following calculations are performed:

**Step-1:** Tables 2, 3, 4 and 5 present professional expert information in the form of *q*-ROHFRS (*q*=3).

**Table 2:** Decision making information.

	$\chi _{1}$	$\chi _{2}$
$A_{1}$	$$\left( \begin{array}{c} \left( \begin{array}{c} \left( 0.1,0.2,0.5\right) , \\ \left( 0.3,0.4\right) \end{array} \right) , \\ \left( \begin{array}{c} \left( 0.8\right) , \\ \left( 0.4,0.6\right) \end{array} \right) \end{array} \right)$$	$$\left( \begin{array}{c} \left( \begin{array}{c} \left( 0.5,0.7\right) , \\ \left( 0.5,0.6\right) \end{array} \right) , \\ \left( \begin{array}{c} \left( 0.4,0.5\right) , \\ \left( 0.7,0.9\right) \end{array} \right) \end{array} \right)$$
$A_{2}$	$$\left( \begin{array}{c} \left( \begin{array}{c} \left( 0.6,0.7\right) , \\ \left( 0.7,0.9\right) \end{array} \right) , \\ \left( \begin{array}{c} \left( 0.3,0.5\right) , \\ \left( 0.6\right) \end{array} \right) \end{array} \right)$$	$$\left( \begin{array}{c} \left( \begin{array}{c} \left( 0.2,0.4,0.5\right) , \\ \left( 0.5\right) \end{array} \right) , \\ \left( \begin{array}{c} \left( 0.6,0.7\right) , \\ \left( 0.3\right) \end{array} \right) \end{array} \right)$$

**Table 3:** Decision making information.

	$\chi _{3}$	$\chi _{4}$
$A_{1}$	$$\left( \begin{array}{c} \left( \begin{array}{c} \left( 0.4\right) , \\ \left( 0.3,0.7\right) \end{array} \right) \mathbf {,} \\ \left( \begin{array}{c} \left( 0.5\right) , \\ \left( 0.9\right) \end{array} \right) \end{array} \right)$$	$$\left( \begin{array}{c} \left( \begin{array}{c} \left( 0.6\right) \mathbf {,} \\ \left( 0.7\right) \end{array} \right) , \\ \left( \begin{array}{c} \left( 0.6,0.8,0.9\right) , \\ \left( 0.7,0.9\right) \end{array} \right) \end{array} \right)$$
$A_{2}$	$$\left( \begin{array}{c} \left( \begin{array}{c} \left( 0.8\right) , \\ \left( 0.4,0.5,0.7\right) \end{array} \right) , \\ \left( \begin{array}{c} \left( 0.2,0.5\right) , \\ \left( 0.4,0.5\right) \end{array} \right) \end{array} \right)$$	$$\left( \begin{array}{c} \left( \begin{array}{c} \left( 0.8\right) , \\ \left( 0.5\right) \end{array} \right) , \\ \left( \begin{array}{c} \left( 0.7\right) , \\ \left( 0.1,0.3,0.4\right) \end{array} \right) \end{array} \right)$$

**Table 4:** Decision making information.

	$\chi _{1}$	$\chi _{2}$
$A_{3}$	$$\left( \begin{array}{c} \left( \begin{array}{c} \left( 0.4,0.5,0.6\right) , \\ \left( 0.6,0.7\right) \end{array} \right) , \\ \left( \begin{array}{c} \left( 0.9\right) , \\ \left( 0.5\right) \end{array} \right) \end{array} \right)$$	$$\left( \begin{array}{c} \left( \begin{array}{c} \left( 0.1\right) , \\ \left( 0.5,0.6\right) \end{array} \right) , \\ \left( \begin{array}{c} \left( 0.4,0.6,0.7\right) , \\ \left( 0.5,0.7\right) \end{array} \right) \end{array} \right)$$
$A_{4}$	$$\left( \begin{array}{c} \left( \begin{array}{c} \left( 0.4\right) , \\ \left( 0.5,0.6\right) \end{array} \right) , \\ \left( \begin{array}{c} \left( 0.3,0.4\right) , \\ \left( 0.8\right) \end{array} \right) \end{array} \right)$$	$$\left( \begin{array}{c} \left( \begin{array}{c} \left( 0.4,0.5\right) , \\ \left( 0.4\right) \end{array} \right) , \\ \left( \begin{array}{c} \left( 0.1,0.2\right) , \\ \left( 0.2,0.3\right) \end{array} \right) \end{array} \right)$$

**Table 5:** Decision making information.

	$\chi _{3}$	$\chi _{4}$
$A_{3}$	$$\left( \begin{array}{c} \left( \begin{array}{c} \left( 0.3\right) , \\ \left( 0.7,0.8\right) \end{array} \right) , \\ \left( \begin{array}{c} \left( 0.7,0.8\right) , \\ \left( 0.1,0.4,0.7\right) \end{array} \right) \end{array} \right)$$	$$\left( \begin{array}{c} \left( \begin{array}{c} \left( 0.3,0.6\right) , \\ \left( 0.8\right) \end{array} \right) , \\ \left( \begin{array}{c} \left( 0.7\right) , \\ \left( 0.3\right) \end{array} \right) \end{array} \right)$$
$A_{4}$	$$\left( \begin{array}{c} \left( \begin{array}{c} \left( 0.3\right) , \\ \left( 0.7,0.8\right) \end{array} \right) , \\ \left( \begin{array}{c} \left( 0.7\right) , \\ \left( 0.6\right) \end{array} \right) \end{array} \right)$$	$$\left( \begin{array}{c} \left( \begin{array}{c} \left( 0.6,0.7,0.9\right) , \\ \left( 0.3,0.4\right) \end{array} \right) , \\ \left( \begin{array}{c} \left( 0.2,0.7\right) , \\ \left( 0.7,0.8,0.9\right) \end{array} \right) \end{array} \right)$$

**Step-2**: The expert information is of benefit type. Therefore, , we need not to normalise the *q*-ROHFR values in this case.
**Step-3**: Only one expert is considered in this problem for the collection of uncertain information. Therefore, we are not required to find the collected information.
**Step-4**: The following information is used to assess the aggregated information for the alternative under the specified set of attributes:

**Case-1:** Table 6 displays aggregation information using *q*-ROHFREWG operator.

**Table 6:** Aggregated information using *q*-ROHFREWG.

$A_{1}$	$$\left( \begin{array}{c} \left( \begin{array}{c} \left\{ 0.0018,0.0025,0.0036,0.0051,0.0090,0.0128\right\} , \\ \left\{ 0.53700.6272,0.9732,0.6466,0.5424,0.6310,0.5689,0.6502\right\} \end{array} \right) \mathbf {,} \\ \left( \begin{array}{c} \left\{ 0.0183,0.0248,0.0284,0.0146,0.0198,0.0226\right\} , \\ \left\{ 0.7662,0.8334,0.8977,0.8759,0.7751,0.8397,0.8326,0.8806\right\} \end{array} \right) \end{array} \right)$$
$A_{2}$	$$\left( \begin{array}{c} \left( \begin{array}{c} \left\{ 0.0120,0.0241,0.0302,0.0120,0.0241,0.0302\right\} , \\ \left\{ 0.5156,0.5366,0.9654,0.59330.6090,0.6608\right\} \end{array} \right) \mathbf {,} \\ \left( \begin{array}{c} \left\{ 0.0038,0.0096,0.0045,0.0113,0.0064,0.0160,0.0075,0.0188\right\} , \\ \left\{ 0.3795,0.3973,0.9836,0.4169,0.4317,0.4512\right\} \end{array} \right) \end{array} \right)$$
$A_{3}$	$$\left( \begin{array}{c} \left( \begin{array}{c} \left\{ 0.0005,0.0011,0.0007,0.0013,0.0008,0.0016\right\} , \\ \left\{ 0.6902,0.7252,0.9973,0.7386,0.7011,0.7347,0.7158,0.7476\right\} \end{array} \right) \mathbf {,} \\ \left( \begin{array}{c} \left\{ 0.0275,0.0317,0.0415,0.0479,0.0488,0.0563\right\} , \\ \left\{ 0.3890,0.5179,0.8973,0.4945,0.5179,0.6048\right\} \end{array} \right) \end{array} \right)$$
$A_{4}$	$$\left( \begin{array}{c} \left( \begin{array}{c} \left\{ 0.0043,0.0051,0.0067,0.0054,0.0063,0.0084\right\} \\ \left\{ 0.5249,0.5382,0.9675,0.59830.53900.55150.59890.6089\right\} , \end{array} \right) \mathbf {,} \\ \left( \begin{array}{c} \left\{ 0.0006,0.0022,0.0013,0.0045,0.0008,0.0030,0.0017,0.0060\right\} , \\ \left\{ 0.62580.6728,0.9843,0.6298,0.6763,0.7409\right\} \end{array} \right) \end{array} \right)$$

**Case-2:** Aggregation information using Einstein ordered weighted averaging operator is shown in Table 7.

**Table 7:** Aggregated information using *q*-ROHFREOWG.

$A_{1}$	$$\left( \begin{array}{c} \left( \begin{array}{c} \left\{ 0.4012,0.4458,0.4376,0.4856,0.4913,0.5440\right\} , \\ \left\{ 0.5240,0.6235,0.5544,0.6446,0.5296,0.6274,0.5594,0.6481\right\} \end{array} \right) \mathbf {,} \\ \left( \begin{array}{c} \left\{ 0.5612,0.5273,0.6107,0.5747,0.6359,0.5990\right\} , \\ \left\{ 0.7713,0.8334,0.8298,0.8759,0.7800,0.8397,0.8362,0.8806\right\} \end{array} \right) \end{array} \right)$$
$A_{2}$	$$\left( \begin{array}{c} \left( \begin{array}{c} \left\{ 0.5183,0.5456,0.6159,0.6464,0.6508,0.6822\right\} , \\ \left\{ 0.5623,0.6992,0.5799,0.7097,0.6372,0.7457\right\} \end{array} \right) \mathbf {,} \\ \left( \begin{array}{c} \left\{ 0.3634,0.4259,0.4721,0.5500,0.3809,0.4461,0.4941,0.5748\right\} , \\ \left\{ 0.4543,0.4812,0.4596,0.4860,0.4671,0.4926\right\} \end{array} \right) \end{array} \right)$$
$A_{3}$	$$\left( \begin{array}{c} \left( \begin{array}{c} \left\{ 0.2220,0.3775,0.2288,0.3889,0.2348,0.3987\right\} , \\ \left\{ 0.6789,0.6972,0.7155,0.7314,0.6904,0.7079,0.7254,0.7406\right\} \end{array} \right) \mathbf {,} \\ \left( \begin{array}{c} \left\{ 0.6180,0.6935,0.7258,0.6451,0.7221,0.7548\right\} , \\ \left\{ 0.3975,0.5116,0.4327,0.5335,0.5482,0.6160\right\} \end{array} \right) \end{array} \right)$$
$A_{4}$	$$\left( \begin{array}{c} \left( \begin{array}{c} \left\{ 0.3889,0.4134,0.3978,0.4228,0.4151,0.4411\right\} \\ \left\{ 0.5451,0.5751,0.6038,0.6279,0.5503,0.5797,0.6079,0.6317\right\} , \end{array} \right) \mathbf {,} \\ \left( \begin{array}{c} \left\{ 0.2749,0.3008,0.3307,0.3616,0.3254,0.3558,0.3908,0.4268\right\} , \\ \left\{ 0.6541,0.6578,0.6728,0.6763,0.7024,0.7055\right\} \end{array} \right) \end{array} \right)$$

**Case-3:** Tables 8 and 9 present the aggregation information using the *q*-ROHFRWG operator.

**Table 8:** Weighted information (EWG).

	$\chi _{1}$	$\chi _{2}$
$A_{1}$	$$\left( \begin{array}{c} \left( \begin{array}{c} \left( 0.8456,0.8902,0.9481\right) , \\ \left( 0.1438,0.1917\right) \end{array} \right) , \\ \left( \begin{array}{c} \left( 0.9801\right) , \\ \left( 0.1917,0.2890\right) \end{array} \right) \end{array} \right)$$	$$\left( \begin{array}{c} \left( \begin{array}{c} \left( 0.8609,0.9217\right) \mathbf {,} \\ \left( 0.3276,0.3944\right) \end{array} \right) , \\ \left( \begin{array}{c} \left( 0.8252,0.8222\right) , \\ \left( 0.4638,0.6331\right) \end{array} \right) \end{array} \right)$$
$A_{2}$	$$\left( \begin{array}{c} \left( \begin{array}{c} \left( 0.9599,0.9703\right) , \\ \left( 0.3400,0.4666\right) \end{array} \right) , \\ \left( \begin{array}{c} \left( 0.9159,0.9481\right) , \\ \left( 0.2890\right) \end{array} \right) \end{array} \right)$$	$$\left( \begin{array}{c} \left( \begin{array}{c} \left( 0.7058,0.8222,0.8609\right) , \\ \left( 0.3276\right) \end{array} \right) , \\ \left( \begin{array}{c} \left( 0.8637,0.8989\right) , \\ \left( 0.1963\right) \end{array} \right) \end{array} \right)$$
$A_{3}$	$$\left( \begin{array}{c} \left( \begin{array}{c} \left( 0.9339,0.9481,0.9599\right) , \\ \left( 0.2890,0.3400\right) \end{array} \right) , \\ \left( \begin{array}{c} \left( 0.9897\right) , \\ \left( 0.2400\right) \end{array} \right) \end{array} \right)$$	$$\left( \begin{array}{c} \left( \begin{array}{c} \left( 0.5970\right) , \\ \left( 0.3276,0.3944\right) \end{array} \right) , \\ \left( \begin{array}{c} \left( 0.8222,0.8933,0.9217\right) , \\ \left( 0.32760.4638\right) \end{array} \right) \end{array} \right)$$
$A_{4}$	$$\left( \begin{array}{c} \left( \begin{array}{c} \left( 0.9339\right) , \\ \left( 0.2400,0.2890\right) \end{array} \right) , \\ \left( \begin{array}{c} \left( 0.9159,0.9339\right) , \\ \left( 0.3960\right) \end{array} \right) \end{array} \right)$$	$$\left( \begin{array}{c} \left( \begin{array}{c} \left( 0.8222,0.8609\right) , \\ \left( 0.2618\right) \end{array} \right) , \\ \left( \begin{array}{c} \left( 0.5970,0.7058\right) , \\ \left( 0.1308,0.1963\right) \end{array} \right) \end{array} \right)$$

**Table 9:** Weighted information (EWG).

	$\chi _{3}$	$\chi _{4}$
$A_{1}$	$$\left( \begin{array}{c} \left( \begin{array}{c} \left( 0.8087\right) , \\ \left( 0.2008,0.4745\right) \end{array} \right) \mathbf {,} \\ \left( \begin{array}{c} \left( 0.8503\right) , \\ \left( 0.6472\right) \end{array} \right) \end{array} \right)$$	$$\left( \begin{array}{c} \left( \begin{array}{c} \left( 0.8811\right) , \\ \left( 0.4797\right) \end{array} \right) , \\ \left( \begin{array}{c} \left( 0.8811,0.9421,0.9705\right) , \\ \left( 0.4797,0.6539\right) \end{array} \right) \end{array} \right)$$
$A_{2}$	$$\left( \begin{array}{c} \left( \begin{array}{c} \left( 0.9440\right) , \\ \left( 0.2679,0.3352,0.4745\right) \end{array} \right) , \\ \left( \begin{array}{c} \left( 0.6846,0.8503\right) , \\ \left( 0.2679,0.3352\right) \end{array} \right) \end{array} \right)$$	$$\left( \begin{array}{c} \left( \begin{array}{c} \left( 0.9421\right) , \\ \left( 0.3389\right) \end{array} \right) , \\ \left( \begin{array}{c} \left( 0.9128\right) , \\ \left( 0.0677,0.2031,0.2708\right) \end{array} \right) \end{array} \right)$$
$A_{3}$	$$\left( \begin{array}{c} \left( \begin{array}{c} \left( 0.7563\right) , \\ \left( 0.4745,0.5518\right) \end{array} \right) , \\ \left( \begin{array}{c} \left( 0.91580.9440\right) , \\ \left( 0.0669,0.2679,0.4745\right) \end{array} \right) \end{array} \right)$$	$$\left( \begin{array}{c} \left( \begin{array}{c} \left( 0.74780.8811\right) , \\ \left( 0.5578\right) \end{array} \right) , \\ \left( \begin{array}{c} \left( 0.9128\right) , \\ \left( 0.2031\right) \end{array} \right) \end{array} \right)$$
$A_{4}$	$$\left( \begin{array}{c} \left( \begin{array}{c} \left( 0.7563\right) , \\ \left( 0.4745,0.5518\right) \end{array} \right) , \\ \left( \begin{array}{c} \left( 0.9158\right) , \\ \left( 0.4036\right) \end{array} \right) \end{array} \right)$$	$$\left( \begin{array}{c} \left( \begin{array}{c} \left( 0.8811,0.9128,0.9705\right) , \\ \left( 0.2031,0.2708\right) \end{array} \right) , \\ \left( \begin{array}{c} \left( 0.6740,0.9128\right) , \\ \left( 0.4797,0.5578,0.6539\right) \end{array} \right) \end{array} \right)$$

The score values of Tables 8 and 9 are presented in Table 10. On the basis of score values ordered the information shown in Tables 11 and 12. Aggregation information using *q*-ROHFREHWG are presented in Table 13.

**Table 10:** Score value of weighted (EWG) matrix.

	$\chi _{1}$	$\chi _{2}$	$\chi _{3}$	$\chi _{4}$
$A_{1}$	0.8667	0.7014	0.6685	0.6915
$A_{2}$	0.8012	0.7884	0.7627	0.8339
$A_{3}$	0.8456	0.6798	0.7258	0.7416
$A_{4}$	0.7996	0.7669	0.6888	0.7285

**Table 11:** Ordered weighted information (EWG).

	$\chi _{1}$	$\chi _{2}$
$A_{1}$	$$\left( \begin{array}{c} \left( \begin{array}{c} \left( 0.8456,0.8902,0.9481\right) , \\ \left( 0.14380.1917\right) \end{array} \right) , \\ \left( \begin{array}{c} \left( 0.9801\right) , \\ \left( 0.1917,0.2890\right) \end{array} \right) \end{array} \right)$$	$$\left( \begin{array}{c} \left( \begin{array}{c} \left( 0.8609,0.9217\right) \mathbf {,} \\ \left( 0.3276,0.3944\right) \end{array} \right) , \\ \left( \begin{array}{c} \left( 0.8252,0.8222\right) , \\ \left( 0.4638,0.6331\right) \end{array} \right) \end{array} \right)$$
$A_{2}$	$$\left( \begin{array}{c} \left( \begin{array}{c} \left( 0.9421\right) , \\ \left( 0.3389\right) \end{array} \right) , \\ \left( \begin{array}{c} \left( 0.9128\right) , \\ \left( 0.0677,0.2031,0.2708\right) \end{array} \right) \end{array} \right)$$	$$\left( \begin{array}{c} \left( \begin{array}{c} \left( 0.9599,0.9703\right) , \\ \left( 0.3400,0.4666\right) \end{array} \right) , \\ \left( \begin{array}{c} \left( 0.9159,0.9481\right) , \\ \left( 0.2890\right) \end{array} \right) \end{array} \right)$$
$A_{3}$	$$\left( \begin{array}{c} \left( \begin{array}{c} \left( 0.9339,0.9481,0.9599\right) , \\ \left( 0.2890,0.3400\right) \end{array} \right) , \\ \left( \begin{array}{c} \left( 0.9897\right) , \\ \left( 0.2400\right) \end{array} \right) \end{array} \right)$$	$$\left( \begin{array}{c} \left( \begin{array}{c} \left( 0.7478,0.8811\right) , \\ \left( 0.5578\right) \end{array} \right) , \\ \left( \begin{array}{c} \left( 0.9128\right) , \\ \left( 0.2031\right) \end{array} \right) \end{array} \right)$$
$A_{4}$	$$\left( \begin{array}{c} \left( \begin{array}{c} \left( 0.9339\right) , \\ \left( 0.2400,0.2890\right) \end{array} \right) , \\ \left( \begin{array}{c} \left( 0.9159,0.9339\right) , \\ \left( 0.3960\right) \end{array} \right) \end{array} \right)$$	$$\left( \begin{array}{c} \left( \begin{array}{c} \left( 0.8222,0.8609\right) , \\ \left( 0.2618\right) \end{array} \right) , \\ \left( \begin{array}{c} \left( 0.5970,0.7058\right) , \\ \left( 0.1308,0.1963\right) \end{array} \right) \end{array} \right)$$

**Table 12:** Ordered weighted information (EWG).

	$\chi _{3}$	$\chi _{4}$
$A_{1}$	$$\left( \begin{array}{c} \left( \begin{array}{c} \left( 0.8811\right) , \\ \left( 0.4797\right) \end{array} \right) \mathbf {,} \\ \left( \begin{array}{c} \left( 0.8811,0.9421,0.9705\right) , \\ \left( 0.4797,0.6539\right) \end{array} \right) \end{array} \right)$$	$$\left( \begin{array}{c} \left( \begin{array}{c} \left( 0.8087\right) , \\ \left( 0.2008,0.4745\right) \end{array} \right) , \\ \left( \begin{array}{c} \left( 0.8503\right) , \\ \left( 0.6472\right) \end{array} \right) \end{array} \right)$$
$A_{2}$	$$\left( \begin{array}{c} \left( \begin{array}{c} \left( 0.70580.82220.8609\right) , \\ \left( 0.3276\right) \end{array} \right) , \\ \left( \begin{array}{c} \left( 0.8637,0.8989\right) , \\ \left( 0.1963\right) \end{array} \right) \end{array} \right)$$	$$\left( \begin{array}{c} \left( \begin{array}{c} \left( 0.9440\right) , \\ \left( 0.2679,0.3352,0.4745\right) \end{array} \right) , \\ \left( \begin{array}{c} \left( 0.6846,0.8503\right) , \\ \left( 0.2679,0.3352\right) \end{array} \right) \end{array} \right)$$
$A_{3}$	$$\left( \begin{array}{c} \left( \begin{array}{c} \left( 0.7563\right) , \\ \left( 0.4745,0.5518\right) \end{array} \right) , \\ \left( \begin{array}{c} \left( 0.9158,0.9440\right) , \\ \left( 0.0669,0.2679,0.4745\right) \end{array} \right) \end{array} \right)$$	$$\left( \begin{array}{c} \left( \begin{array}{c} \left( 0.5970\right) , \\ \left( 0.3276,0.3944\right) \end{array} \right) , \\ \left( \begin{array}{c} \left( 0.8222,0.8933,0.9217\right) , \\ \left( 0.3276,0.4638\right) \end{array} \right) \end{array} \right)$$
$A_{4}$	$$\left( \begin{array}{c} \left( \begin{array}{c} \left( 0.8811,0.9128,0.9705\right) , \\ \left( 0.2031,0.2708\right) \end{array} \right) , \\ \left( \begin{array}{c} \left( 0.6740,0.9128\right) , \\ \left( 0.4797,0.5578,0.6539\right) \end{array} \right) \end{array} \right)$$	$$\left( \begin{array}{c} \left( \begin{array}{c} \left( 0.7563\right) , \\ \left( 0.4745,0.5518\right) \end{array} \right) , \\ \left( \begin{array}{c} \left( 0.9158\right) , \\ \left( 0.4036\right) \end{array} \right) \end{array} \right)$$

**Table 13:** Aggregated information using *q*-ROHFREHWG.

$A_{1}$	$$\left( \begin{array}{c} \left( \begin{array}{c} \left\{ 0.8487,0.8655,0.8546,0.8713,0.8624,0.8791\right\} , \\ \left\{ 0.3544,0.4220,0.3722,0.4348,0.3558,0.4229,0.3735,0.4357\right\} \end{array} \right) \mathbf {,} \\ \left( \begin{array}{c} \left\{ 0.8702,0.8884,0.8972,0.8694,0.8876,0.8964\right\} , \\ \left\{ 0.5257,0.5798,0.5719,0.6179,0.5283,0.5819,0.5741,0.6197\right\} \end{array} \right) \end{array} \right)$$
$A_{2}$	$$\left( \begin{array}{c} \left( \begin{array}{c} \left\{ 0.8814,0.9140,0.9247,0.8844,0.9170,0.9277\right\} , \\ \left\{ 0.3169,0.3348,0.3894,0.3652,0.3789,0.4234\right\} \end{array} \right) \mathbf {,} \\ \left( \begin{array}{c} \left\{ 0.8292,0.8803,0.8396,0.8905,0.8384,0.8894,0.8488,0.8995\right\} , \\ \left\{ 0.2450,0.2734,0.2507,0.2780,0.2584,0.2843\right\} \end{array} \right) \end{array} \right)$$
$A_{3}$	$$\left( \begin{array}{c} \left( \begin{array}{c} \left\{ 0.7261,0.7620,0.7280,0.7640,0.7297,0.7657\right\} , \\ \left\{ 0.4518,0.4646,0.4793,0.4907,0.4550,0.4676,0.4821,0.4934\right\} \end{array} \right) \mathbf {,} \\ \left( \begin{array}{c} \left\{ 0.8968,0.9181,0.9268,0.9052,0.9264,0.9350\right\} , \\ \left\{ 0.2470,0.3277,0.2738,0.3438,0.3584,0.4043\right\} \end{array} \right) \end{array} \right)$$
$A_{4}$	$$\left( \begin{array}{c} \left( \begin{array}{c} \left\{ 0.8339,0.8434,0.8614,0.8444,0.8539,0.8718\right\} \\ \left\{ 0.3484,0.3949,0.3573,0.4019,0.3520,0.3977,0.3608,0.4046\right\} , \end{array} \right) \mathbf {,} \\ \left( \begin{array}{c} \left\{ 0.7581,0.8283,0.7892,0.8593,0.7605,0.8308,0.7917,0.8617\right\} , \\ \left\{ 0.3940,0.4305,0.4819,0.3971,0.4330,0.4840\right\} \end{array} \right) \end{array} \right)$$

**Step-5**: Table 14 shows the score values for all alternatives under established aggregation operators.

**Table 14:** Score values.

Operators	$SR\left( A_{1}\right)$	$SR\left( A_{2}\right)$	$SR\left( A_{3}\right)$	$SR\left( A_{4}\right)$
*q*-ROHFREWG	0.4989	0.5066	0.5018	0.4867
*q*-ROHFREOWG	0.4082	0.4860	0.4460	0.3727
*q*-ROHFREHWG	0.6943	0.7849	0.7163	0.7119
**Step-6**: Table 15 illustrates the ranking of the alternatives $A_k(k=1,2,\ldots .,4).$

**Table 15:** The ranking of the alternatives.

Operators	Score	Best Alternative
*q*-ROHFREWG	$SR\left( A_{2}\right)>SR\left( A_{3}\right)>SR\left( A_{1}\right) >SR\left( A_{4}\right)$	$A_{2}$
*q*-ROHFREOWG	$SR\left( A_{2}\right)>SR\left( A_{3}\right)>SR\left( A_{1}\right) >SR\left( A_{4}\right)$	$A_{2}$
*q*-ROHFREHWG	$SR\left( A_{2}\right)>SR\left( A_{3}\right)>SR\left( A_{4}\right) >SR\left( A_{1}\right)$	$A_{2}$

**Figure 1:**
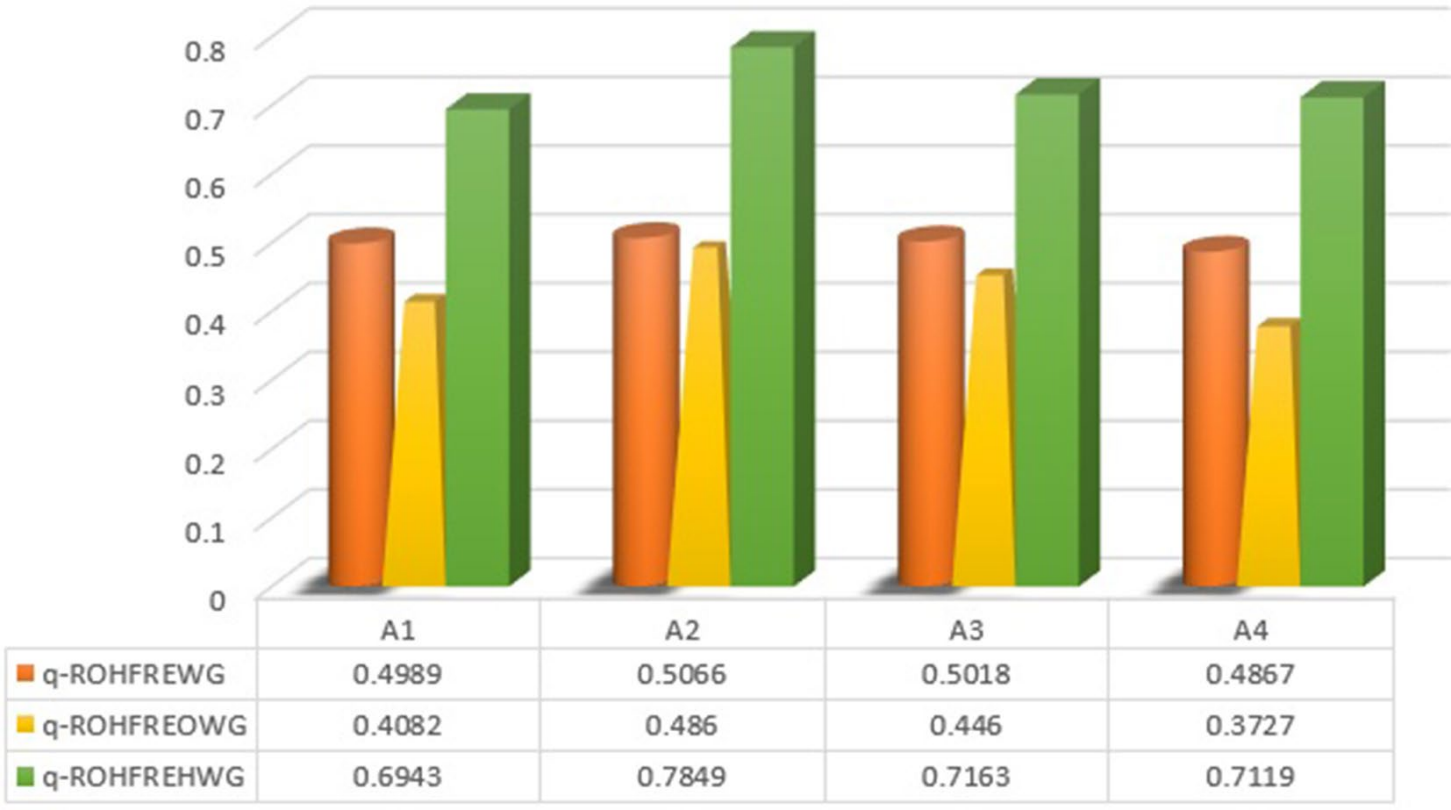
The graphical representation of ranking under proposed operators.

We determined that alternative $A_{2}$ is the best choice among the others based on the findings of the prior computational technique and so strongly recommend it. The graphical representation of alternatives are depicted in Fig. 1.

## Comparison analysis

To demonstrate the characteristics of the developed technique clearly, we shall perform a comparison with TOPSIS approach.

### The TOPSIS approach utilizing *q*-ROHFR information

Hwang and Yoon^11^ introduced the TOPSIS approach for evaluating ideal solutions, which enables policymakers to evaluate ideal positive and negative solutions. TOPSIS is predicated on the idea that the optimal alternative is the one that is closest to the positive ideal solution and furthest from the negative ideal solution^10,14^. Through the following steps, we will develop an approach for ranking all of the alternatives using improved TOPSIS technique:

Firstly, let $A =\{A _{1},A _{2},A _{3},\ldots ,A _{m}\}$ be the set of alternatives and $C=\{\chi _{1},\chi _{2},\chi _{3},\ldots ,\chi _{n}\}$ be a set of criteria. The expert’s decision matrix is as follows:

$$\begin{aligned} M= & {} \left[ \underline{\mathcal {Y} }(\mu _{ij}^{\hat{\jmath }}),\overline{\mathcal {Y} } (\mu _{ij}^{\hat{\jmath }})\right] _{m\times n} \\= & {} \left[ \begin{array}{cccc} \left( \underline{\mathcal {Y} }(\varrho _{11}),\overline{\mathcal {Y} }(\varrho _{11})\right) &{} \left( \underline{\mathcal {Y} }(\varrho _{12}),\overline{\mathcal {Y} }(\varrho _{12})\right) &{} \cdots &{} \left( \underline{\mathcal {Y} }(\varrho _{1j}), \overline{\mathcal {Y} }(\varrho _{1j})\right) \\ \left( \underline{\mathcal {Y} }(\varrho _{21}),\overline{\mathcal {Y} }(\varrho _{21})\right) &{} \left( \underline{\mathcal {Y} }(\varrho _{22}),\overline{\mathcal {Y} }(\varrho _{22})\right) &{} \cdots &{} \left( \underline{\mathcal {Y} }(\varrho _{2j}), \overline{\mathcal {Y} }(\varrho _{2j})\right) \\ \left( \underline{\mathcal {Y} }(\varrho _{31}),\overline{\mathcal {Y} }(\varrho _{31})\right) &{} \left( \underline{\mathcal {Y} }(\varrho _{32}),\overline{\mathcal {Y} }(\varrho _{32})\right) &{} \cdots &{} \left( \underline{\mathcal {Y} }(\varrho _{3j}), \overline{\mathcal {Y} }(\varrho _{3j})\right) \\ \vdots &{} \vdots &{} \ddots &{} \vdots \\ \left( \underline{\mathcal {Y} }(\varrho _{i1}),\overline{\mathcal {Y} }(\varrho _{i1})\right) &{} \left( \underline{\mathcal {Y} }(\varrho _{i2}),\overline{\mathcal {Y} }(\varrho _{i2})\right) &{} \cdots &{} \left( \underline{\mathcal {Y} }(\varrho _{ij}), \overline{\mathcal {Y} }(\varrho _{ij})\right) \end{array} \right] , \end{aligned}$$

where

$$\begin{aligned} \overline{\mathcal {Y} }(\varrho _{ij})=\left\{ \left\langle \mu ,\eth _{h_{\overline{ \mathcal {Y} }(\varrho )}}(\mu ),\psi _{h_{\overline{\mathcal {Y} }(\varrho )}}(\mu )\right\rangle |\mu \in \pounds \right\} \end{aligned}$$

and

$$\begin{aligned} \underline{\mathcal {Y} }(\varrho )=\left\{ \left\langle \mu ,\eth _{h_{\underline{ \mathcal {Y} }(\varrho )}}(\mu ),\psi _{h_{\underline{\mathcal {Y} }(\varrho )}}(\mu )\right\rangle |\mu \in \pounds \right\} \end{aligned}$$

such that

$$\begin{aligned} 0\le \left( \max (\eth _{h_{\overline{\mathcal {Y} }(\varrho )}}(\mu ))\right) ^{q}+\left( \min (\psi _{h_{\overline{\mathcal {Y} }(\varrho )}}(\mu ))\right) ^{q}\le 1 \end{aligned}$$

and

$$\begin{aligned} 0\le \left( \min (\eth _{h_{\underline{\mathcal {Y} }(\varrho )}}(\mu )\right) ^{q}+\left( \max (\psi _{h_{\underline{\mathcal {Y} }(\varrho )}}(\mu ))\right) ^{q}\le 1 \end{aligned}$$

are the *q*-ROHF rough values. Secondly, we collect information from DMs in the form of *q*-ROHFRNs.

Thirdly, normalise the data defined by DMs, since the decision matrix may include both benefits and cost criteria, as illustrated below:

$$\begin{aligned} \left( H\right) ^{_{\hat{\jmath }}}=\left[ \begin{array}{cccc} \left( \overline{\mathcal {Y} }(\varrho _{11}^{_{\hat{\jmath }}}),\underline{\mathcal {Y} } (\varrho _{11}^{_{\hat{\jmath }}})\right) &{} \left( \overline{\mathcal {Y} }(\varrho _{12}^{_{\hat{\jmath }}}),\underline{\mathcal {Y} }(\varrho _{12}^{_{\hat{\jmath } }})\right) &{} \cdots &{} \left( \overline{\mathcal {Y} }(\varrho _{1j}^{_{\hat{ \jmath }}}),\underline{\mathcal {Y} }(\varrho _{1j}^{_{\hat{\jmath }}})\right) \\ \left( \overline{\mathcal {Y} }(\varrho _{21}^{_{\hat{\jmath }}}),\underline{\mathcal {Y} } (\varrho _{21}^{_{\hat{\jmath }}})\right) &{} \left( \overline{\mathcal {Y} }(\varrho _{22}^{_{\hat{\jmath }}}),\underline{\mathcal {Y} }(\varrho _{22}^{_{\hat{\jmath } }})\right) &{} \cdots &{} \left( \overline{\mathcal {Y} }(\varrho _{2j}^{_{\hat{ \jmath }}}),\underline{\mathcal {Y} }(\varrho _{2j}^{_{\hat{\jmath }}})\right) \\ \left( \overline{\mathcal {Y} }(\varrho _{31}^{_{\hat{\jmath }}}),\underline{\mathcal {Y} } (\varrho _{31}^{_{\hat{\jmath }}})\right) &{} \left( \overline{\mathcal {Y} }(\varrho _{32}^{_{\hat{\jmath }}}),\underline{\mathcal {Y} }(\varrho _{32}^{_{\hat{\jmath } }})\right) &{} \cdots &{} \left( \overline{\mathcal {Y} }(\varrho _{3j}^{_{\hat{ \jmath }}}),\underline{\mathcal {Y} }(\varrho _{3j}^{_{\hat{\jmath }}})\right) \\ \vdots &{} \vdots &{} \ddots &{} \vdots \\ \left( \overline{\mathcal {Y} }(\varrho _{i1}^{_{\hat{\jmath }}}),\underline{\mathcal {Y} } (\varrho _{i1}^{_{\hat{\jmath }}})\right) &{} \left( \overline{\mathcal {Y} }(\varrho _{i2}^{_{\hat{\jmath }}}),\underline{\mathcal {Y} }(\varrho _{i2}^{_{\hat{\jmath } }})\right) &{} \cdots &{} \left( \overline{\mathcal {Y} }(\varrho _{ij}^{_{\hat{ \jmath }}}),\underline{\mathcal {Y} }(\varrho _{ij}^{_{\hat{\jmath }}})\right) \end{array} \right] , \end{aligned}$$

where $\hat{\jmath }$ identifies the number of experts.

Fourthly, assess the normalised matrices of experts $\left( N\right) ^{\hat{ \jmath }},$ as

$$\begin{aligned} \left( N\right) ^{\hat{\jmath }}=\left\{ \begin{array}{ccc} \mathcal {Y} (\varrho _{ij})=\left( \underline{\mathcal {Y} }\left( \varrho _{ij}\right) , \overline{\mathcal {Y} }\left( \varrho _{ij}\right) \right) &{} \text {if} &{} \text {For benefit} \\ \left( \mathcal {Y} (\varrho _{ij})\right) ^{c}=\left( \left( \underline{\mathcal {Y} }\left( \varrho _{ij}\right) \right) ^{c},\left( \overline{\mathcal {Y} }\left( \varrho _{ij}\right) \right) ^{c}\right) &{} \text {if} &{} \text {For cost } \end{array} \right. \end{aligned}$$

Fifthly, determine the positive ideal solution and the negative ideal solution based on the score value. Positive ideal solutions and negative ideal solutions are represented as: $\Upsilon ^{+}=\left( \Gamma _{1}^{+},\Gamma _{2}^{+},\Gamma _{3}^{+},\ldots ,\Gamma _{n}^{+}\right)$ and $\Upsilon ^{-}=\left( \Gamma _{1}^{-},\Gamma _{2}^{-},\Gamma _{3}^{-},\ldots ,\Gamma _{n}^{-}\right)$ respectively. For positive ideal solution $\Upsilon ^{+}$, it can be calculated as follows:

$$\begin{aligned} \Upsilon ^{+}= & {} \left( \Gamma _{1}^{+},\Gamma _{2}^{+},\Gamma _{3}^{+},\ldots ,\Gamma _{n}^{+}\right) \\= & {} \left( \max _{i}score(\Gamma _{i1}),\max _{i}score\Gamma _{i2},\max _{i}score\Gamma _{i3},\ldots ,\max _{i}score\Gamma _{in}.\right) \end{aligned}$$

Similarly, the following formula may be used to find the negative ideal solution:

$$\begin{aligned} \Upsilon ^{-}= & {} \left( \Gamma _{1}^{-},\Gamma _{2}^{-},\Gamma _{3}^{-},\ldots \Gamma _{n}^{-}\right) \\= & {} \left( \min _{i}score\Gamma _{i1},\min _{i}score\Gamma _{i2},\min _{i}score\Gamma _{i3},\ldots ,\min _{i}score\Gamma _{in}\right) . \end{aligned}$$

Afterwards, determine the geometric distance between all possible options and the positive ideal $\Upsilon ^{+}$ as follows:

$$\begin{aligned} d(\alpha _{ij},\Upsilon ^{+})= & {} \frac{1}{8}\left( \begin{array}{c} \left( \begin{array}{c} \frac{1}{\#h}\sum _{s=1}^{\#h}\left| \left( \underline{\mu } _{ij(s)}\right) ^{2}-\left( \underline{\mu }_{i}^{+}\right) ^{2}\right| \\ +\left| \left( \overline{\mu }_{ij(s)}\right) ^{2}-\left( \overline{\mu } _{i(s)}^{+}\right) ^{2}\right| \end{array} \right) \\ +\left( \begin{array}{c} \frac{1}{\#g}\sum _{s=1}^{\#g}\left| \left( \underline{\delta } _{ij(s)}\right) ^{2}-\left( \underline{\delta }_{i(s)}^{+}\right) ^{2}\right| \\ +\left| \left( \overline{\delta _{h}}_{_{ij}}\right) ^{2}-\left( \overline{ \delta _{h}}_{_{i}}^{+}\right) ^{2}\right| \end{array} \right) \end{array} \right) ,\text { } \\ \text {where }i= & {} 1,2,3,\ldots ,n,\text {and }j=1,2,3,\ldots ,m. \end{aligned}$$

Likewise, the geometric distance between all possible alternatives and the negative ideal $\Upsilon ^{-}$ can be find as follows:

$$\begin{aligned} d(\alpha _{ij},\Upsilon ^{-})= & {} \frac{1}{8}\left( \begin{array}{c} \left( \begin{array}{c} \frac{1}{\#h}\sum _{s=1}^{\#h}\left| \left( \underline{\mu } _{ij(s)}\right) ^{2}-\left( \underline{\mu }_{i(s)}^{-}\right) ^{2}\right| \\ +\left| \left( \overline{\mu }_{ij(s)}\right) ^{2}-\left( \overline{\mu } _{i(s)}^{-}\right) ^{2}\right| \end{array} \right) \\ +\left( \begin{array}{c} \frac{1}{\#g}\sum _{s=1}^{\#g}\left| \left( \underline{\delta } _{ij(s)}\right) ^{2}-\left( \underline{\delta }_{i(s)}^{-}\right) ^{2}\right| \\ +\left| \left( \overline{\delta _{h}}_{_{ij}}\right) ^{2}-\left( \overline{ \delta _{h}}_{_{i}}^{-}\right) ^{2}\right| \end{array} \right) \end{array} \right) , \\ \text {where }i= & {} 1,2,3,\ldots ,n,\text {and } j=1,2,3,\ldots ,m. \end{aligned}$$

Sixthly, the following formula is used to determine the relative closeness indices for all decision makers of the alternatives:

$$\begin{aligned} RC(\alpha _{ij})=\frac{d(\alpha _{ij},\Upsilon ^{+})}{d(\alpha _{ij},\Upsilon ^{-})+d(\alpha _{ij},\Upsilon ^{+})} \end{aligned}$$

Finally, determine the ranking order of alternatives, and then choose the most desirable alternative that is the smallest distance.

### Numerical example

Through a numerical example, this section will describe the features and validity of the suggested approach for selecting a wind power plant location.

**Step-1**: Tables 2, 3, 4 and 5 contain information regarding decision makers in the form of *q*-ROHFRNs.
**Step-2**: Table 16 computes both positive and negative ideal solutions as follows:

**Table 16:** Ideal solutions.

Criteria	$\Upsilon ^{+}$	$\Upsilon ^{-}$
$\chi _{1}$	$$\left( \begin{array}{c} \left( \begin{array}{c} \left( 0.4,0.5,0.6\right) , \\ \left( 0.6,0.7\right) \end{array} \right) , \\ \left( \begin{array}{c} \left( 0.9\right) , \\ \left( 0.5\right) \end{array} \right) \end{array} \right)$$	$$\left( \begin{array}{c} \left( \begin{array}{c} \left( 0.4\right) , \\ \left( 0.5,0.6\right) \end{array} \right) , \\ \left( \begin{array}{c} \left( 0.3,0.4\right) , \\ \left( 0.8\right) \end{array} \right) \end{array} \right)$$
$\chi _{2}$	$$\left( \begin{array}{c} \left( \begin{array}{c} \left( 0.2,0.4,0.5\right) , \\ \left( 0.5\right) \end{array} \right) , \\ \left( \begin{array}{c} \left( 0.6,0.7\right) , \\ \left( 0.3\right) \end{array} \right) \end{array} \right)$$	$$\left( \begin{array}{c} \left( \begin{array}{c} \left( 0.1\right) , \\ \left( 0.5,0.6\right) \end{array} \right) , \\ \left( \begin{array}{c} \left( 0.4,0.6,0.7\right) , \\ \left( 0.5,0.7\right) \end{array} \right) \end{array} \right)$$
$\chi _{3}$	$$\left( \begin{array}{c} \left( \begin{array}{c} \left( 0.8\right) , \\ \left( 0.4,0.5,0.7\right) \end{array} \right) , \\ \left( \begin{array}{c} \left( 0.2,0.5\right) , \\ \left( 0.4,0.5\right) \end{array} \right) \end{array} \right)$$	$$\left( \begin{array}{c} \left( \begin{array}{c} \left( 0.4\right) , \\ \left( 0.3,0.7\right) \end{array} \right) \mathbf {,} \\ \left( \begin{array}{c} \left( 0.5\right) , \\ \left( 0.9\right) \end{array} \right) \end{array} \right)$$
$\chi _{4}$	$$\left( \begin{array}{c} \left( \begin{array}{c} \left( 0.8\right) , \\ \left( 0.5\right) \end{array} \right) , \\ \left( \begin{array}{c} \left( 0.7\right) , \\ \left( 0.1,0.3,0.4\right) \end{array} \right) \end{array} \right)$$	$$\left( \begin{array}{c} \left( \begin{array}{c} \left( 0.6\right) \mathbf {,} \\ \left( 0.7\right) \end{array} \right) , \\ \left( \begin{array}{c} \left( 0.6,0.8,0.9\right) , \\ \left( 0.7,0.9\right) \end{array} \right) \end{array} \right)$$

**Step-3**: Determine the distance between the positive and negative ideal solutions.

**Table:** 

0.6019	0.2140	0.3849	0.5427

and

**Table:** 

0.3265	0.5313	0.5035	0.4266

**Step-4**: The following are the relative closeness indices for DMs of the alternatives:

**Table:** 

$A_{1}$	$A_{2}$	$A_{3}$	$A_{4}$
0.6483	0.2871	0.4333	0.5599
**Step-5**: According to the aforementioned findings and Fig. 2, $A_2$ has the shortest distance. As a consequence, $A_2$ is the most appropriate option. By synthesizing the above concepts, we can conclude that proposed solution based on *q*-ROHFRSs is effective and reasonable for handling MCDM problems.

**Figure 2:**
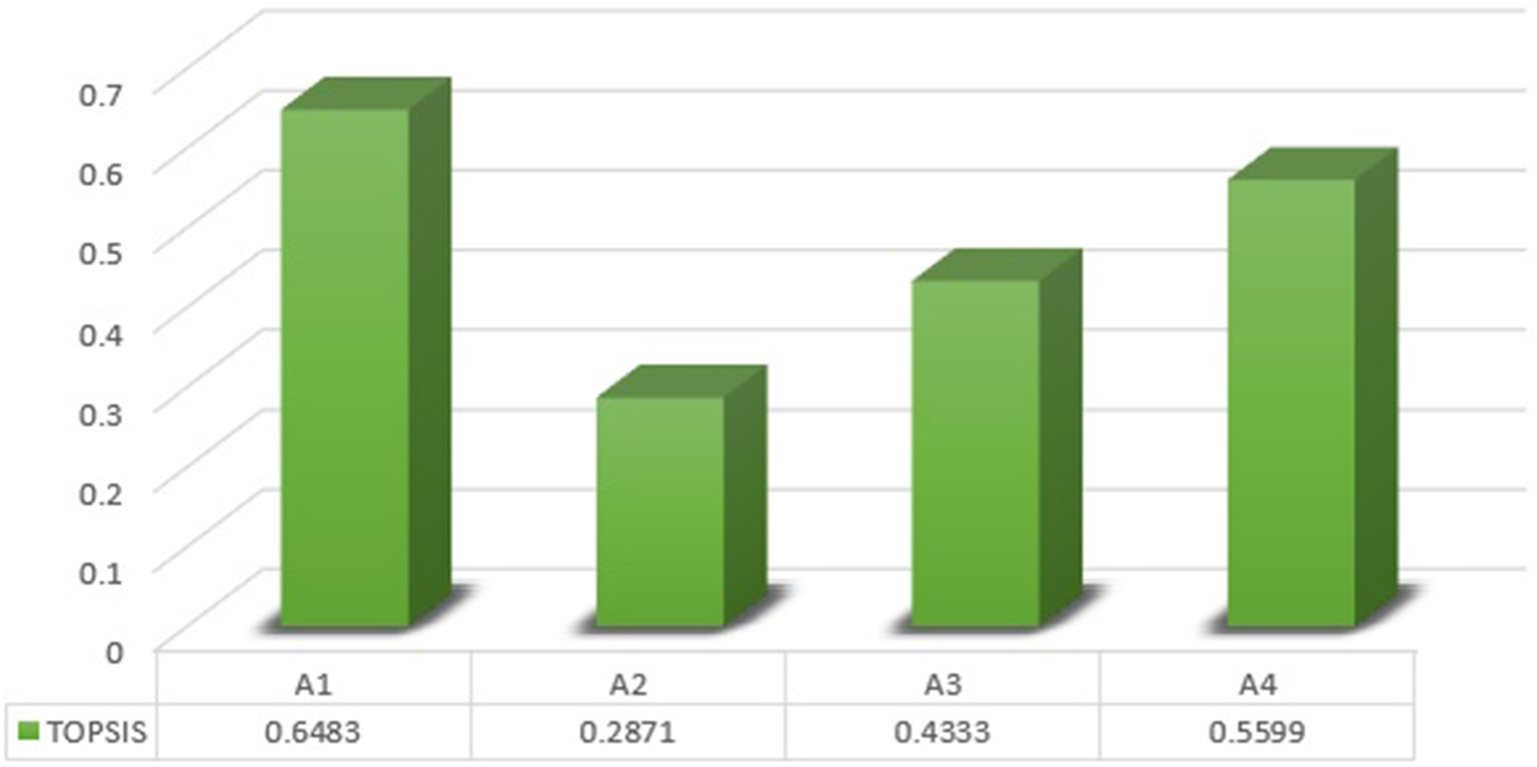
The graphical representation of ranking under TOPSIS method.

## Conclusion

Choosing the best site for establishing the projects is a crucial stage in wind energy power stations. There are several aspects to consider while deciding the best location for the plants to be installed, which is a significant stage in wind energy projects. Therefore, a novel approach based on *q*-ROHFRS is suggested for assessment in order to overcome the restrictions and support the researcher in selecting an appropriate site for installing a wind power station. The knowledge of the concepts presented in this study provide a broad space for analyzing information, enabling decision-makers to incorporate the features of uncertain data and having a high computing capabilities for uncertain information. A list of geometric aggregation operators is presented based on the proposed approach, employing Einstein’s t-norm and t-conorm. The aforementioned methodology can handle the complication of the MADM approach based on the *q*-ROHFRS, and the evaluation information is very reasonable. Furthermore, a case study in the evaluation of wind power plant site selection schemes together with comparative analysis using the improved *q*-ROHFR-TOPSIS approach demonstrates the approach’s validity and reasonability. In the future, the established approach can be extended to other fuzzy and uncertain situations such as language and probability sets to broaden the space for representation of analysing information, adapt to a wider range of evaluation environments, and improve the method’s flexibility. Additionally, within the context of three-way notions, it is worthwhile to investigate consensus procedures based on *q*-ROHFRS.
