# Author Correction: A wind power plant site selection algorithm based on *q*-rung orthopair hesitant fuzzy rough Einstein aggregation information

**DOI:** 10.1038/s41598-022-11165-0

**Published:** 2022-04-28

**Authors:** Attaullah, Shahzaib Ashraf, Noor Rehman, Asghar Khan, Muhammad Naeem, Choonkil Park

**Affiliations:** 1 Department of Mathematics, Abdul Wali Khan University, Mardan, 23200 KPK Pakistan; 2 Department of Mathematics, Khawaja Farid University of Engineering and Information Technology, Rahim Yar Khan, Pakistan; 3 Department of Mathematics and Statistics, Bacha Khan University, Charsadda, KPK Pakistan; 4 Deanship of Combined First Year, Umm Al-Qura University, Makkah, Saudi Arabia; 5 Research Institute for Natural Sciences, Hanyang University, Seoul, Korea

Correction to: *Scientific Reports* 10.1038/s41598-022-09323-5, published online 31 March 2022

The original version of this Article contained errors.

Shahzaib Ashraf was incorrectly affiliated with ‘Department of Mathematics and Statistics, Bacha Khan University, Charsadda, KPK, Pakistan’. The correct affiliation is listed below.

Department of Mathematics, Khawaja Farid University of Engineering and Information Technology, Rahim Yar Khan, Pakistan

In the Acknowledgements section,

“Muhammad Naeem would like to thank the Deanship of Scientific Research at Umm Al-Qura University for supporting this work by Grant Code: (22UQU4310396DSR02).”

now reads:

“Muhammad Naeem would like to thank the Deanship of Scientific Research at Umm Al-Qura University for supporting this work by Grant Code: (22UQU4310396DSR06).”

The original Article has been corrected.

